# Overexpression of ABA Receptor *PYL10* Gene Confers Drought and Cold Tolerance to Indica Rice

**DOI:** 10.3389/fpls.2019.01488

**Published:** 2019-11-28

**Authors:** Rakesh Kumar Verma, Vinjamuri Venkata Santosh Kumar, Shashank Kumar Yadav, Suchitra Pushkar, Mandali Venkateswara Rao, Viswanathan Chinnusamy

**Affiliations:** ^1^Division of Plant Physiology, ICAR–Indian Agricultural Research Institute, New Delhi, India; ^2^Department of Plant Science, School of Life Sciences, Bharathidasan University, Tiruchirappalli, India

**Keywords:** abscisic acid, membrane stability, relative water content, transgenic rice, yield

## Abstract

Abscisic acid (ABA) plays versatile functions in regulating plant development and tolerance to various biotic and abiotic stresses. Towards elucidating the functions of one of the ABA receptors (ABARs) in rice, *OsPYL10* was cloned from drought tolerant rice cv. Nagina 22 and was overexpressed under stress inducible *RD29A* promoter in a mega rice variety MTU1010 by using Agrobacterium mediated genetic transformation. Four single copy transgenic lines selected based on Southern blot analysis were used for physiological and molecular analysis. PYL10 receptor appears to regulate its ligand ABA accumulation as *PYL10* overexpressing transgenics accumulated 2–3.3-fold higher levels of ABA than that of WT in flag leaf at anthesis under non-stress conditions. The enhanced accumulation of ABA was associated with enhanced expression of genes for ABA biosynthesis viz., *ZEP1*, *NCED1*, *NCED2*, *NCED3*, and *NCED4* in transgenics than in WT plants. At seedling stage, *PYL10* transgenics showed significantly higher survival rate under cold stress as compared with WT plants. qRT-PCR analysis showed that expression levels of cold responsive genes viz., *DREB1F*, *MYB3R2*, *TPP1*, *COR410*, *DEHYDRIN*, *and LEA3* were significantly higher in PYL10 overexpressing transgenic lines as compared to WT plants under cold stress. *PYL10* transgenic and WT plants grown in the same pot were subjected to -80 kPa drought stress and recovery treatments at vegetative and reproductive stages. At vegetative stage drought stress, three overexpressing lines showed significantly higher grain yield (40–58%) and at reproductive stage drought stress one of these overexpression lines showed two-fold higher grain yield than that of WT plants. Excised leaf water loss analysis showed that *PYL10* transgenic lost about 20% less water than WT plants. At reproductive stage, *OsPYL10* transgenic maintained higher RWC, membrane stability index, chlorophyll content, and accumulated lower amount of MDA and H_2_O_2_ as compared with WT plants. qRT-PCR analysis showed that expression levels of *RAB16*, *Dehydrin*, *LEA3*, and *ABA45* were higher in *PYL10* transgenics as compared with WT plants under drought stress. Thus, overall results showed that *OsPYL10* overexpression has potential to improve both drought and cold stress tolerance of indica rice.

## Introduction

Abscisic acid (ABA), a classical plant hormone, is involved in regulation of several physiological processes from germination to seed development and responses to various environmental stresses (Leung and Giraudat, 1998; Zhu, 2002; Shinozaki and Yamaguchi-Shinozaki, 2007; Finkelstein, 2013). ABA induces root growth for water mining, stomatal closure for transpiration minimization/water conservation, and expression of genes involved in osmoregulation, osmoprotection, and stress damage control and repair under drought and other abiotic stress conditions to impart stress tolerance (Verslues and Bray, 2006; Shinozaki and Yamaguchi- Shinozaki 2007; Finkelstein, 2013; Zhu, 2016). Since 1980s, significant efforts have been made to discover ABA receptors, which culminated in the discovery of PYL (Pyrabactin Resistance1-like)/RCAR (REGULATORY COMPONENTS OF ABA RECEPTORS) family of ABA receptors in 2009 (Ma et al., 2009; Park et al., 2009) and elucidation of signalling pathway and structural mechanisms (Fujii et al., 2009; Melcher et al., 2009; Miyazono et al., 2009; Nishimura et al., 2009; Santiago et al., 2009; Yin et al., 2009; Peterson et al., 2010). In Arabidopsis, PYL/RCAR family consists of 14 members and belongs to a SART domain super family of lipophilic ligand-binding proteins. When ABA is not bound, PYR1, PYL1, and PYL2 form homodimers, while upon binding to ABA all members becomes monomers. Upon binding of ABA to the ligand-binding pocket of PYL protein, the SGLPA ‘gate’ loop and the HRL ‘latch’ loop undergo significant conformational changes which close the ligand-binding pocket. In the ligand-bound confirmation of PYL, the tryptophan residue (lock) of the clade A PP2C is inserted between the gate and latch loops and form PYL-ABA-PP2C complex which inhibits the activity of PP2Cs (Melcher et al., 2009; Miyazono et al., 2009; Santiago et al., 2009; Nishimura et al., 2009; Yin et al., 2009; Peterson et al., 2010). The IC50 values for different PYL-PP2C combinations varied between 18–390 nM (+)-ABA. Different PYLs have different affinity to different PP2Cs (Ma et al., 2009; Nishimura et al., 2009; Park et al., 2009; Santiago et al., 2009; Yin et al., 2009; Peterson et al., 2010). In the absence of ABA binding to PYL receptors, PP2Cs interact with and inhibit the activity of ABA-regulated SnRK2 family kinases viz., SnRK2.2, SnRK2.3, and SnRK2.6. In the presence of ABA, PYL receptor binds to ABA, interacts with PP2Cs and inhibits the activity of PP2Cs, and thus relieves the repression of SnRK2 activity by PP2Cs. SnRK2 kinases phosphorylate their target proteins to regulate stomatal closure and gene expression (Weiner et al., 2010; Zhu, 2016).

Since the discovery of PYL family of ABA receptors in Arabidopsis, ABA receptors have been identified in different crops and efforts are being made to functionally validate the ABA receptors by overexpression and mutational approaches (**Table 1**).

**Table 1:** A list of ABA receptors functionally validated in plants.

S.No.	Gene	Source	Promoter	Transgenic plant	Phenotype and stress tolerance	Reference
1	*OsPYL5*	Arabidopsis	CaMV35S, Overexpression	Arabidopsis	Drought tolerance at seedling stage	Santiago et al., 2009
2	*AtPYL8*	Arabidopsis	CaMV35S, Overexpression	Arabidopsis	ABA hypersensitivity at seedling stage; Drought tolerance at seedling stage	Lim et al., 2013
3	*AtPYL9*	Arabidopsis	RD29A, Overexpression	Arabidopsis and Rice	Drought tolerance and enhanced leaf senescence at seedling stage in both Arabidopsis and rice	Zhao et al., 2016
4	*RCAR11/12/13/14*	Arabidopsis	CaMV35S, Overexpression	Arabidopsis	ABA hypersensitivity at germination and post germination; Drought Tolerance at seedling stage	Li et al., 2018
5	*AtPYL5*	Arabidopsis	pGC1, Overexpression	Arabidopsis	Drought stress tolerance at vegetative stage	Quan et al., 2018
6	*RCAR6, RCAR10*	Arabidopsis	CaMV35S, Overexpression	Arabidopsis	Modulate plant water use efficiency and water productivity in seedlings	Yang et al., 2019
7	*RCAR6/PYL12*	Arabidopsis	CaMV35S, Overexpression	Arabidopsis	Increased water use efficiency with higher growth rate in seedlings	Yang et al., 2016
8	*VlPYL1*	*Vitis labrusca*	CaMV35S, Overexpression	Arabidopsis	Enhances ABA sensitivity at germination and seedling stage	Gao et al., 2018
9	*SlPYL*	Tomato	CaMV35S, Overexpression	Arabidopsis	Drought resistance at seedling stage	González-Guzmán et al., 2014
10	*BrPYL1*	*Brassica rapa*	CaMV35S, Overexpression	Arabidopsis	ABA hypersensitivity during seed germination	Li et al., 2017
11	*GhPYL9-11A*	Cotton	CaMV35S, Overexpression	Arabidopsis	hypersensitive to ABA during seed germination and early seedling stage	Liang et al., 2017
12	*GhPYL10/12/26*	Cotton	CaMV35S, Overexpression	Arabidopsis	Increased ABA sensitivity during germination and early seedling growth and Drought tolerance at flowering stage	Chen et al., 2017
13	*ZmPYL8/9/12*	Maize	CaMV35S, Overexpression	Arabidopsis	Increase ABA sensitivity at germination stage, Drought Resistance at seedling stage	He et al., 2018
14	*FePYR1*	*Festuca elata*	CaMV35S, Overexpression	Arabidopsis	Enhanced drought tolerance at seedlings stage	Ren et al., 2017
15	*MpPYL1*	*Marchantia polymorpha*	MpEF1, Overexpression	Liverworts	ABA-hypersensitive growth with enhanced desiccation tolerance at seedling stage	Jahan et al., 2019
16	*PtPYRL1*, *PtPYRL5*	Poplar	CaMV35S, Overexpression	Arabidopsis	Abscisic Acid Sensitivity at germination and Drought Resistance at seedling stage	Yu et al., 2016
17	*AaPYL9*	*Artemisia annua*	CaMV35S, Overexpression	Arabidopsis	Enhances drought tolerance and improves Artemisinin content at vegetative stage	Zhang et al., 2013
18	*FaPYR1*	*Strawberry*	RNAi	Strawberry	Involve in fruit ripening at maturity	Chai et al., 2011
19	*TaPYL*	Wheat	Overexpression	Wheat	Enhanced water-use efficiency and drought tolerance at flowering stage	Mega et al., 2019
20	*OsPYL8, OsPYL9*	Rice	ZmUbq, Overexpression	Arabidopsis	hypersensitivity to ABA during seed germination	Chen et al., 2017
21	*OsPYL3*	Rice	CaMV35S, Overexpression	Arabidopsis	Enhances Cold and Drought Tolerance at seedling stage	Lenka et al., 2018
22	*OsPYL/RCAR5*	Rice	ZmUbq, Overexpression	Rice	Hypersensitive to ABA during seed germination and early seedling growth	Kim et al., 2012
23	*OsPYL/RCAR5*	Rice	ZmUbq, Overexpression	Rice	Enhances drought and salt stress tolerance at seedling stage	Kim et al., 2014
24	*OsPYL3*, *OsPYL9*	Rice	CaMV35S, Overexpression	Rice	Improved drought and cold stress tolerance at seedling stage	Tian et al., 2015
25	*OsPYL1/4/6*	Rice	CRISPR/Cas9	Rice	Decreases the ABA sensitivity at germination and improves grain yield under non-stress conditions	Miao et al., 2018

Bioinformatic analysis showed that rice genome encodes 10-13 PYL family ABA receptors (Kim et al., 2012; He et al., 2014; Tian et al., 2015; Miao et al., 2018). Biochemical characterization by using recombinant OsPYL1, OsPYL2, OsPYL3, OsPYL6, OsPYL10, OsPYL11, and OsPYL12 from in rice showed that OsPYL1, OsPYL2, and OsPYL3 are dimers (He et al., 2014). The members of ABA receptor family in rice is differently named in different earlier studies (**Supplementary Table 1**) (Kim et al., 2012; He et al., 2014; Tian et al., 2015; Miao et al., 2018). The functions of some members of PYL family in rice have been earlier analyzed by transgenic overexpression and knockout mutants. Constitutive overexpression of *OsRCAR5*/*OsPYL5* driven by maize Ubiquitin promoter in transgenic rice resulted in ABA hypersensitive germination and seedling growth (Kim et al., 2012). Further it conferred tolerance to drought and salt tolerance at vegetative stage in rice. However, constitutive overexpression of *OsPYL5* resulted in reduction in plant height and yield under non-stress conditions (Kim et al., 2014). Constitutive overexpression of *OsPYL3* and *OsPYL9* under the transcriptional control of CaMV35S promoter in rice showed that these genes confer ABA-hypersensitivity during germination and tolerance to drought and cold stresses at vegetative stage (Tian et al., 2015). We showed that constitutive overexpression of *OsPYL3* in Arabidopsis confers improved cold and drought stress tolerance to transgenic Arabidopsis (Lenka et al., 2018). According to the recent nomenclature (Miao et al., 2018), *OsPYL3* is renamed as *OsPYL10*. In this study, we further analyzed the function of *OsPLY10* (= *OsPYL3*, Lenka et al., 2018) in transgenic indica rice mega variety MTU1010. We further analyzed the ABA levels and expression of *OsPYL10* and several stress responsive genes under different stress conditions to elucidate the function of OsPYL10.

## Materials and Methods

### Expression Analysis of *OsPYL10* in Rice

Rice genotype Nagina 22 (*Oryza sativa* ssp. indica cv. Nagina 22) was used for analysis of tissue specific and stress responsive expression of *OsPYL10*. *OsPYL10* expression was analyzed in different tissues from plants at anthesis stage exposed to control and drought stress treatments. At seedling stage, plants grown in Yosida’s medium (YM) were treated with YM supplemented with 20% PEG 6000 (-0.49 MPa), 200 mM NaCl (-1.01 MPa; 20 dS/m) and cold (4°C) stresses and 100 µM ABA for 6 h. Samples were collected from control, and treated plants and frozen in liquid nitrogen. ABA receptors expression in different developmental stages were analyzed using public database Genevestigator and stress specific expression of *OsPYL10* were retrieved from rice array database (www.ricearray.org).

### Construction of *OsPYL10* Over-Expression Vector

Full-length cDNA fragment (615bp) of *OsPYL10* (LOC_Os05g15640) was PCR amplified from cDNA prepared from drought tolerant rice (cv. Nagina22) using proof reading Hotstart DNA polymerase (Kapa, Biosystem, US) and confirmed by sequencing (GenBank: KF925265.1). *OsPYL10* CDS was cloned in modified plant transformation vectors *pCAMBIA1300* under stress inducible promoter *AtRD29A* (**Supplementary Figure 3**). Binary construct was confirmed by colony PCR, restriction digestion, and DNA sequencing. Confirmed clone was transformed in to Agrobacterium strain EHA105 and used for genetic transformation of rice.

### Genetic Transformation of Rice

Rice transformation was performed following the Agrobacterium-mediated co-cultivation method (Sahoo et al., 2011) with some modifications. Seeds of indica rice variety MTU1010 were dehusked and sterilised with 70% ethanol for 90 s followed by 2% NaOCl for 20 min. Sterilized seeds were air dried in a laminar flow hood for 2 h and then placed on callus induction media (CIM). The CIM is MS media containing casein hydrolase (300 mg/L), proline (560 mg/L), maltose (36 g/L), 2-4-D (2.5 mg/L), BAP (0.25 mg/L), and Phytagel (3 g/L) with a PH of 5.8. Two-week old embryogenic calli were used for Agrobacterium-mediated transformation. Primary Agrobacterium culture was grown in YEM medium for 12 h. Calli were infected with Agrobacterium culture and kept in co-cultivation media for 48 h under dark condition at 28°C. After extensive washing, the calli were kept on selection medium for 45 days under dark at 28°C on a MS medium containing hygromycin (50 mg/L) and cefotaxime (250 mg/L). Afterwards, the calli were transferred on to MS medium containing 50 mg/L hygromycin and 250 mg/L cefotaxime for an additional 20 days under light at 28°C. Actively growing calli were then transferred to regeneration medium and cultured under light at 28°C. This resulted in a 17–19% regeneration frequency followed by rooting and hardening. The plants were grown till maturity in a transgenic greenhouse and seeds were harvested.

### Molecular Confirmation of Transgenic Rice Plants

Genomic DNA isolated from the leaf samples of WT and putative transgenic plants was used as template for PCR confirmation with primers *OsPYL10*, *HPTII*, *RD29A*, and *NOS* combinations using Taq DNA Polymerase (Invitrogen, US). 10 µg purified DNA from PCR-confirmed transgenics and WT plants were digested with *Bam*HI, separated in 0.8% agarose gel and capillary transferred onto a nylon membrane for Southern hybridization. *HPTII* probe was prepared with DIG labelling following the user manual of the DIG DNA Labelling and Detection kit (Roche Applied Science, US). Southern hybridization was done following standard protocol.

### Germination Assay

ABA sensitivity during germination was assayed using *OsPYL10* transgenic and WT seeds. Seeds were dehusked, sterilized and were kept in MS media supplemented with 2 µM ABA. Germination was recorded daily till the maximum germination of WT seeds. Germination assay was performed with three biological repetitions.

### Evaluation for Cold and Drought Tolerance of *OsPYL10* Transgenic Rice Lines

#### Cold Stress Treatment

Cold stress tolerance in *OsPYL10* transgenic and WT was evaluated at seedling stage. Seedlings were grown in soilrite in small pots in culture room at 32°C under 16 h light/8 h dark cycle for 25 days. For cold stress treatment, seedlings were shifted to a cold chamber at 15°C for 2 days followed by 6°C for 2 days with three biological replications. Recovery was done at 32°C for a week and survival percent and biomass were recorded.

#### Drought Stress Treatment

Transgenic lines were germinated on MS medium supplemented with hygromycin. Hygromycin resistant seedlings were transferred to soil rite and a week after, well grown transgenic *OsPYL10* and WT seedlings were transplanted in soil filled pots with at least three biological repetitions. In each pot, one transgenic and one WT plants were transplanted to ensure equal drought stress imposition to both WT and transgenic lines. Plants were grown at about 30±2°C and 60–70% RH in transgenic green house, IARI, New Delhi. For one set of plants, drought stress was imposed at vegetative stage, while for other set of plants drought stress was imposed at reproductive stage by with-holding water till the soil matric potential (SMP) reached upto -80 kPa. Physiological and biochemical data were recorded from *OsPYL10* and WT plants under irrigated and drought stress conditions. Leaf samples were also store at -80°C for gene expression analyses. Recovery was done by rewatering and plants were grown till maturity to record biomass and yield data. Three biological replicates were used for recording all the observations.

### Measurement of Physiological and Biochemical Traits

In drought stress experiments, SMP was measured in pots by using tensiometer (Empl, India) and moisture content was measured by gravimetric method. Plant water status was assessed in terms relative water content measured in the top most fully expanded/flag leaf (Barrs and Weatherley, 1962). Total chlorophyll content was measured following the method of Hiscox and Israelstam, 1979. Membrane stability was measured following the protocol of Blum and Ebercon 1981. Photosynthesis was measured in topmost fully expanded/flag leaf by using IRGA (LiCOR6400, USA). MDA and H_2_O_2_ contents of the control and stress plants were determined following the methods described earlier (Heath and Packer, 1968; Alexieva et al., 2001). ABA levels in the flag leaf of WT and PYL10 transgenic lines were quantified following HPLC method (Zeevart, 1980) with three biological repetitions.

### qRT-PCR Analysis of Gene Expression

Total RNA was isolated (Plant RNA mini kit, Invitrogen, US) and quantified using NanoDrop (Thermo Fisher, US). 2 µg of total RNA from each sample was used for cDNA preparation using reverse transcriptase enzyme (Superscript III^RT^, Invitrogen, US). cDNA was used as template for real time PCR with Kapa Hotstart sybergreen master mix. PCR conditions were 95°C for 30 s, followed by 40 cycles of 95°C for 5 s and 60°C for 40 s on Real Time PCR (ABI, Life Technology, USA).

Expression of ABA regulated genes (*OsRAB16*, *OsLEA3*, *ABA45*, and *OsDEHYDRIN*) and genes for ABA metabolism (*OsNCED1*, *OsNCED2*, *OsNCED3*, *OsNCED4*, *OsNCED5*, *OsABAox3*, *OsZEP*, and *OsBG2*) were carried out with the samples collected from reproductive stage drought stress. *AtRD29A* promoter induction to measure *PYL10-NOS* transcript levels in transgenic rice at 22°C and 32°C was performed with T-DNA specific primers *OsPYL10q-F* and *NOS-R*.

Analysis of cold stress responsive genes *OsDREB1A*, *OsDREB1B*, *OsDREB1F*, *OsMYB3R2*, *OsTPP1*, *OsWRKY76*, *OsCOR410*, *OsDEHYDRIN*, and *OsLEA3* were carried out with the samples collected from cold treated plants (15°C for 48 h followed by 6°C for 48 h). *UBIQUITIN5* gene was used as reference gene. All primers used for *OsPYL10* gene cloning and qRT-PCR are listed in **Supplementary Tables 2** and **3**.

### Statistical Analysis

One-way ANOVA was carried out for analysis of variation for significance in mean value. The R package software (R studio) used for analysis of variance for gene expression data and alphabets denote the variation. *mean *P < 0.05*.

## Results and Discussion

### Expression of *OsPYL10* in Rice

**Figure 1:**
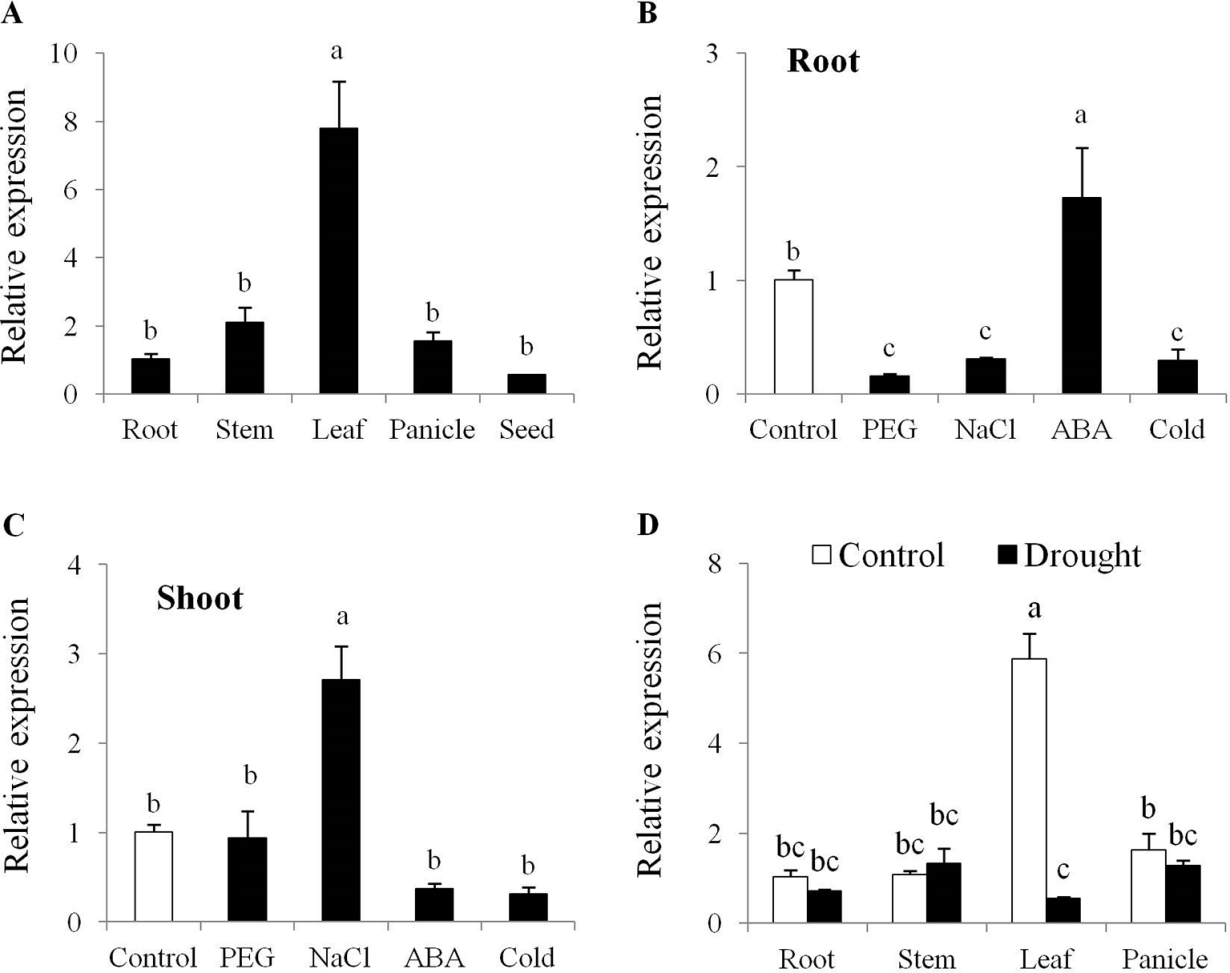
Tissue specific and stress responsive expression of *OsPYL10 gene* in rice cv. Nagina 22 **(A)** *OsPYL10* gene expression in different tissues at flowering stage under control conditions. **(B**–**C)** *OsPYL10* gene expression under different stress conditions. 14 days old Nagina 22 seedlings were treated with PEG6000 (20%), NaCl (200 mM), ABA (100 µM) and cold (4°C) for 6 hours and expression of *OsPYL10* gene was analyzed in root **(B)** and shoot **(C)** tissues. **(D)** *OsPYL10* gene expression in different tissues at flowering stage under control and drought conditions in the field. Sample were collected for RNA isolation from field under control and drought (-80kPa; Soil moisture content = 7.6%) conditions. Value shown in graph are mean of three biological replicate (n = 3). Error bar indicates ±SE. Statistical differences are shown on the bars by labelling significantly different groups with different letters.

Tissue specific expression analysis showed that *OsPYL10* expression was highest in leaf tissues as compared with root, stem, panicle, and seed under non-stress conditions (**Figure 1A**) and was significantly downregulated in leaf under drought conditions (**Figure 1D**). In seedling stage, ABA and NaCl treatments upregulated the expression of *PYL10* in root and shoot tissue, respectively. PEG and cold stresses significantly downregulated the expression of *PYL10* in roots (**Figures 1B, C**). Expression analysis of *OsPYL10* at different developmental stages of rice using Genevestigator database revealed that *OsPYL10* expresses at all developmental stages with highest expression at milk and dough stages (**Supplementary Figure 1A**). Rice array database revealed that *OsPYL10* expression was downregulated under dehydration in all growth stages and highest induction was found in NaCl stress treatment in leaves (**Supplementary Figure 1B**).

### Development *PYL10* Transgenic Rice Lines

**Figure 2:**
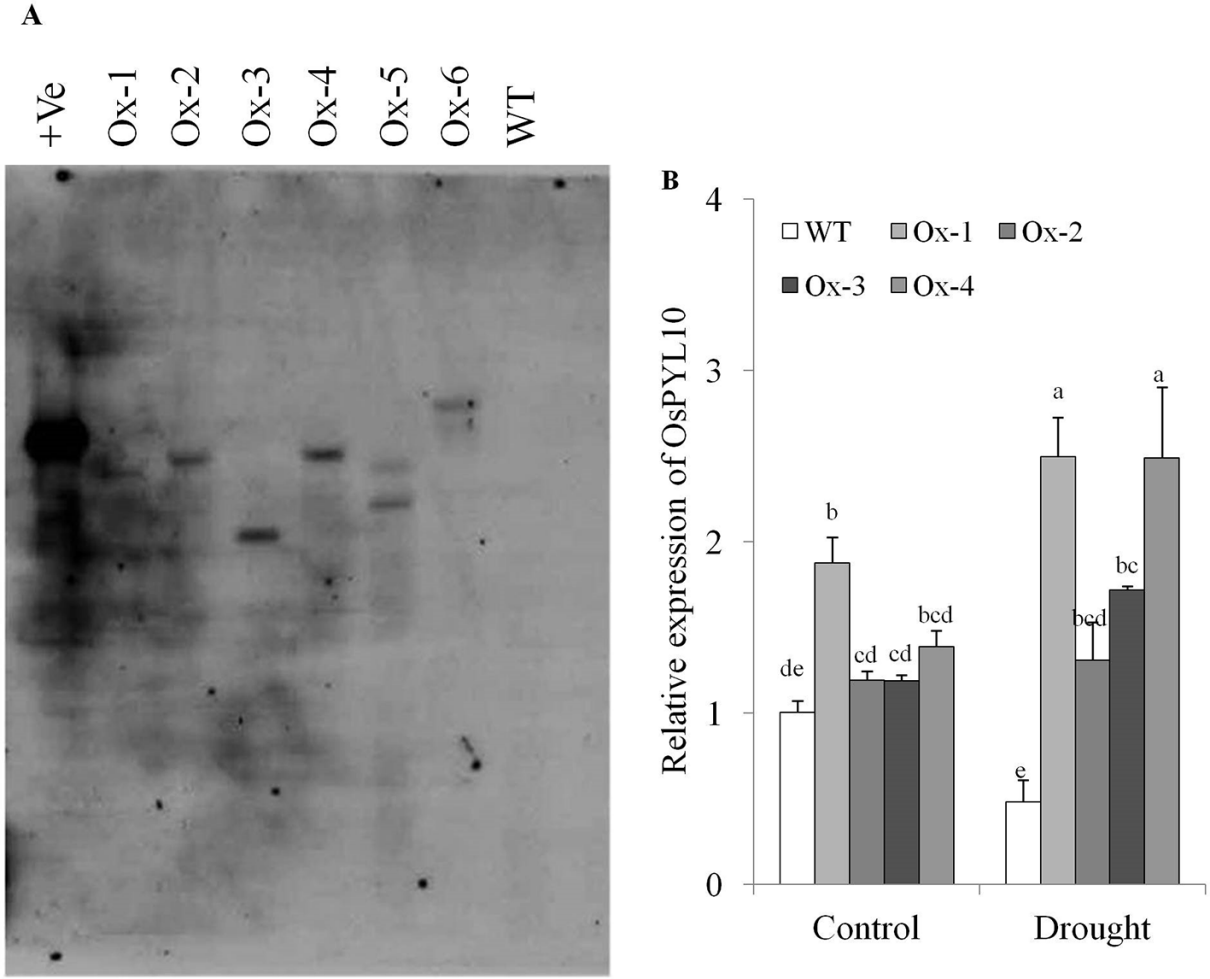
Molecular confirmation of transgenic lines **(A)** Southern blot analysis of overexpressing *OsPYL10* transgenic lines (T1). DNA was extracted from transgenic lines (*Ox1*, *Ox2*, *Ox3*, *Ox4*, *Ox5* and *Ox6*) and WT plants. 10µg DNA was overnight digested with restriction enzyme (*KpnI)* and run in 1x TAE gel for 5 h at 50 volt and transferred in nitrocellulose membrane and hybridized with *HPTII* probe labelled with DIG and autoradiogram was developed. **(B)** qRT-PCR analysis for relative gene expression in *OsPYL10* transgenic lines under control and drought stress conditions. Samples were collected for RNA isolation from control and drought stress (-80kPa; Soil moisture content = 7.6%) conditions. Values shown in graph are mean of three biological replicates (n = 3). Error bar indicates ±SE. Statistical differences are shown on the bars by labelling significantly different groups with different letters.

We cloned OsPYL10 (=*OsPYL3/RCAR3*, Kim et al., 2012) from rice cv. Nagina 22. Full length CDS (615 bp) was cloned and sequenced (NCBI GeneBank Acc. No. KF925265). PYL10 CDS was cloned under the transcriptional control of stress inducible promoter *AtRD29A* promoter in pCAMBIA1300 vector and confirmed by restriction digestion and DNA sequencing. Binary construct was transformed into Agrobacterium strain *EHA105* (**Supplementary Figures S3A, B**). Rice cv. MTU1010 was transformed and six transgenic lines (Ox-1, Ox-2, Ox-3, Ox-4, Ox-5, and Ox-6) were generated. These transgenic lines were confirmed by PCR with T-DNA specific primers viz., *HPTII* and *OsPYL10-F*, *and Nos-R* combinations (**Supplementary Figure 2C**). Further, Southern blot analysis at T1 generation showed that transgenic lines Ox-1, Ox-2, Ox-3, and Ox-4 have single integration while Ox-5 and Ox-6 have two copies of integration (**Figure 2A**). Hence we used events Ox1, Ox2, Ox3, and Ox4 in all the experiments, except for qRT-PCR analysis of genes for ABA metabolism, where two representative events (Ox3 and Ox4) were analyzed. qRT-PCR expression analysis *OsPYL10* levels in flag leaf showed that *PYL10* expression in transgenics was statistically similar to that of WT except for *Ox-1* transgenic line which showed significantly higher expression levels. However, under drought stress conditions, all single copy transgenic lines studied showed significantly higher expression levels over WT plants (**Figure 2B**).

### *OsPYL10* Overexpression Confers Enhanced ABA Sensitivity

Overexpression of hormone receptor is expected to enhance the sensitivity of the transgenic plant to the hormone in respective bioassay. ABA sensitivity at germination was used as bioassay to examine the *PYL10* transgenics. WT and *PYL10* transgenic (T1) seeds harvested at the same time were dried and germinated on MS media supplemented with 0 or 2 µM ABA. In the absence of ABA, both WT and PYL10 germinated and grew similarly. In the absence of ABA, WT, and PYL10 transgenic lines reached 100% germination by 4 days. However, addition of 2 µM ABA to the growth medium significantly inhibited germination with WT plants showed 80% germination, while PYL10 overexpressing lines showed less than 32–57% germination by 4^th^ day (**Supplementary Figures 4A–C**).

### *OsPYL10* Modulates the ABA Content through Regulation of Genes for ABA Metabolism

**Figure 3:**
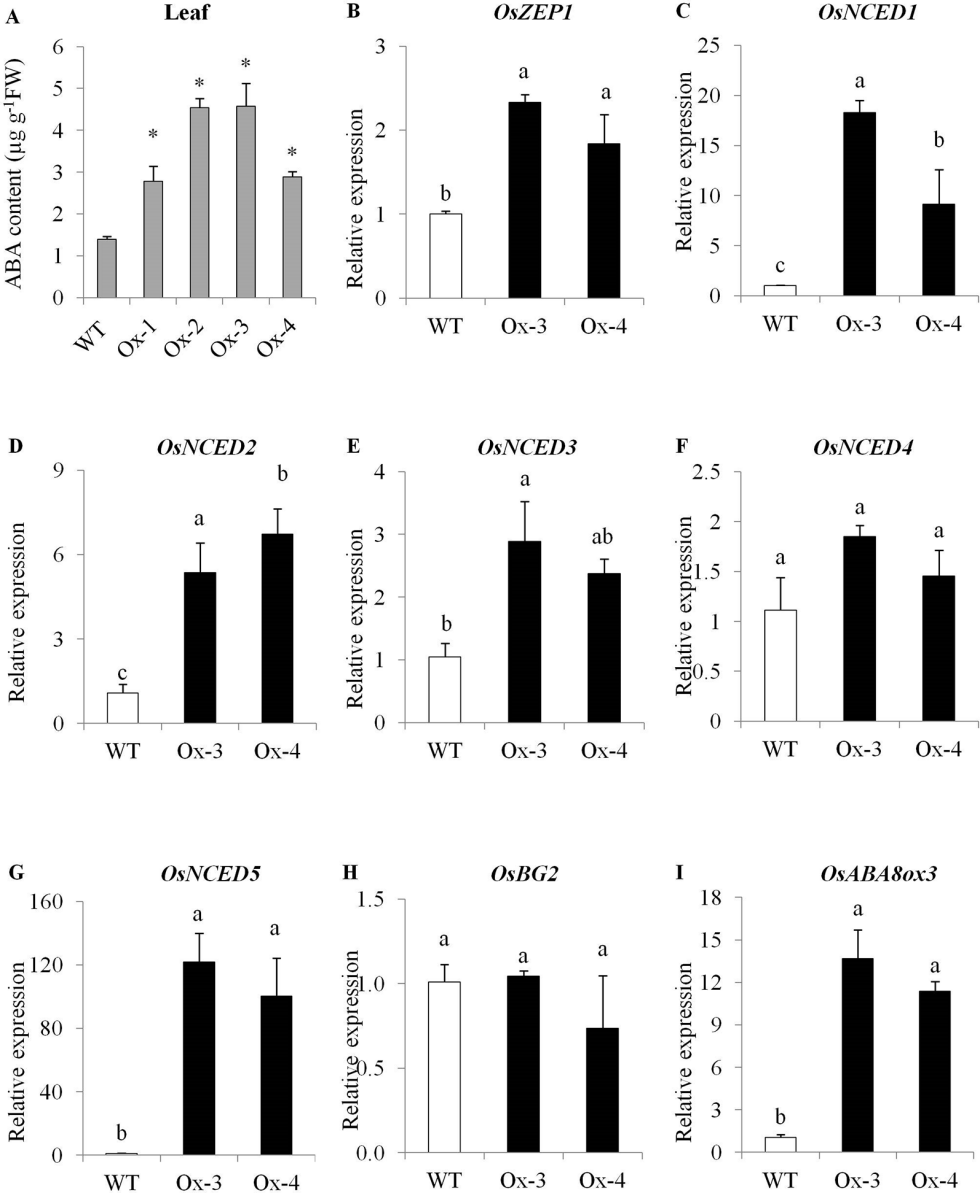
PYL10 regulates ABA content and genes for ABA metabolism. **(A)** PYL10 overexpression enhances ABA content in transgenic rice. **(B**–**I)** qRT-PCR gene expression of genes for ABA biosynthesis viz., *OsZEP1*, *OsNCED1*, *OsNCED2*, *OsNCED3* and *OsNCED5*, ABA catabolism (*OsABA8ox3*) and ABA conjugation (*OsBG2*). Flag leaf sample from about 15 independent plants (biological samples) for each transgenic event were collected at flowering stage under control conditions. The leaves were cut in to small pieces and three replicates of about 1g each was sampled for three replicates for each transgenic event, and ABA was quantified by HPLC method. Part of the same flag leaf sample was used for gene expression analysis. The experiments were performed with three biological replicates. Values are mean (n = 3). Error bar indicates ±SE. Statistical differences are shown on the bars by labelling significantly different groups with different letters. *means *P* < 0.05.

Under non-stress conditions, flag leaves of WT plants at flowering stage accumulated about 1.4 µg ABA g^-1^ fresh weight, while transgenic plants accumulated 2–3-fold higher levels of ABA as compared with WT plants (**Figure 3A**). Since we used stress inducible *AtRD29A* promoter to drive transgene (*OsPYL10*) expression, change in ABA levels under normal conditions was unexpected. To understand constitutively high levels of ABA in transgenic lines, we examined *AtRD29A* promoter in the transgenic rice lines by qRT-PCR analysis. We examined *RD29A* promoter induction in transgenic rice at 22°C (a growth temperature conducive for Arabidopsis) and 32°C (where rice plants are gown, optimum for rice). We found that transcripts of PYL10-NOS (OsPYL10 fused with NOS terminator) accumulated to a higher levels at 32°C as compared to that under 22°C in transgenic lines. This suggests that *AtRD29A* promoter is induced and transgene *PYL10* transcripts accumulate to certain level even at 32°C at which rice plants are grown normally (**Supplementary Figure S5**). This might have caused altered sensitivity of the transgenic plants to the normal levels of ABA, and in turn caused ABA accumulation. To understand the causes of enhanced ABA levels, the expression levels of ABA biosynthetic and catabolic pathway genes was analyzed using qRT-PCR. Since Ox1 and Ox4 showed similar ABA levels, and Ox2 and Ox3 showed similar ABA levels, we selected representative Ox3 and Ox4 for expression analysis of genes for ABA metabolism. PYL10 overexpression lines Ox-3 and Ox-4 showed significantly higher expression levels of ABA biosynthesis genes viz., *OsZEP1*, *OsNCED1*, *OsNCED2*, *OsNCED3*, and *Os-NCED5* as compared with WT plants. Among the catabolic genes, *OsBG2* expression was unaltered, while *OsABA8ox3* gene expression was significantly higher in transgenic lines over WT plants (**Figures 3B–I**).

### *OsPYL10* Confers Cold Stress Tolerance

**Figure 4:**
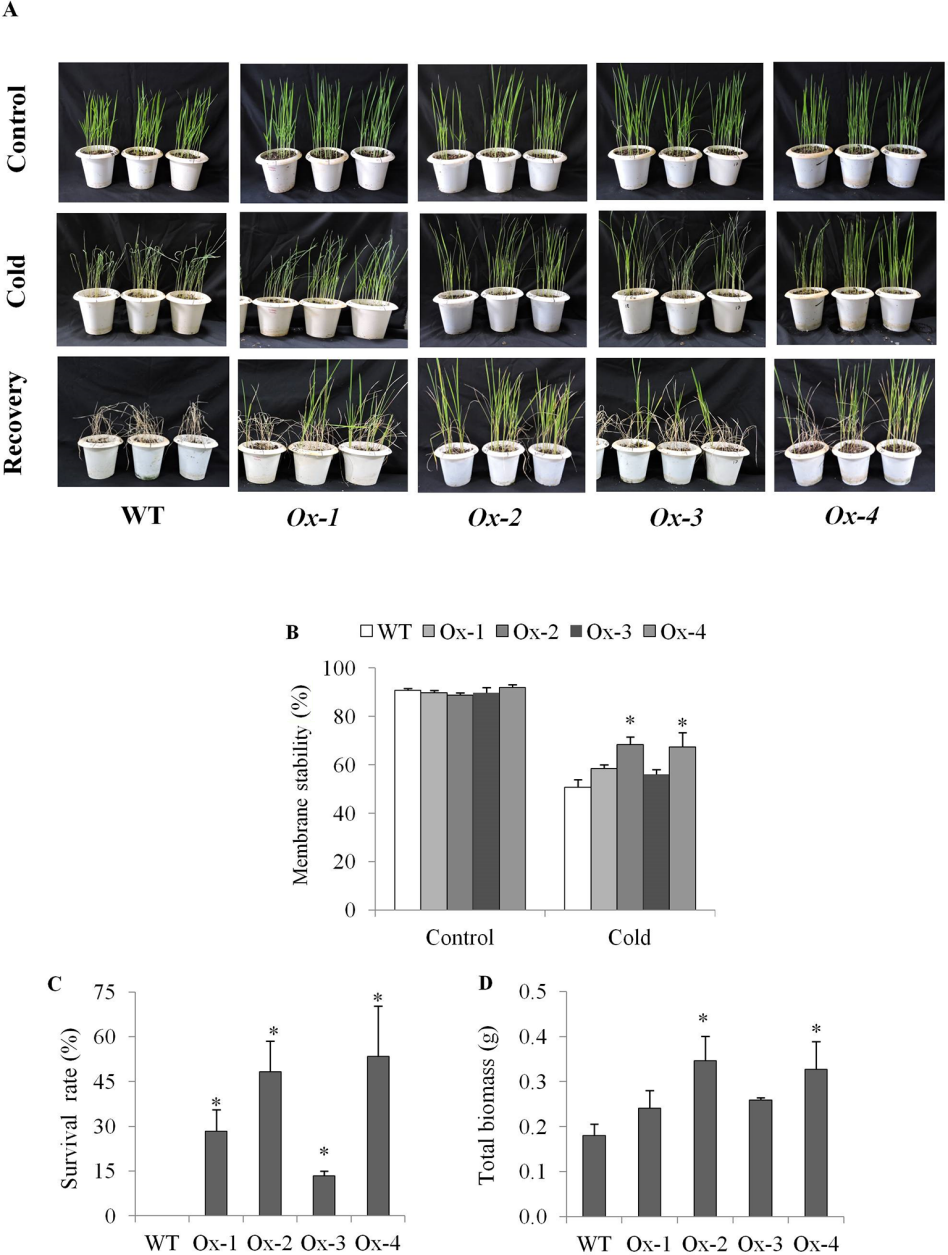
Cold stress tolerance of *OsPYL10* overexpressing transgenic rice seedlings. **(A)** Photograph showing phenotype of seedling before and after stress and recovery. 25 days old seedlings were exposed to 15°C for 48 h (acclimation) followed by cold stress at 6°C for 48 h. These plants were recovered at normal temperature for one week and then photographs were taken. **(B)** Membrane stability at the end of cold stress treatment. **(C**, **D)** Seedling survival rate and total biomass after 7 days of cold stress recovery. The experiments were performed with three independent biological replicates (3 pots each with 20 seedlings). Value shown in graph are mean of biological replicates (n = 20). Error bar indicates ±SE. *means *P* < 0.05.

**Figure 5:**
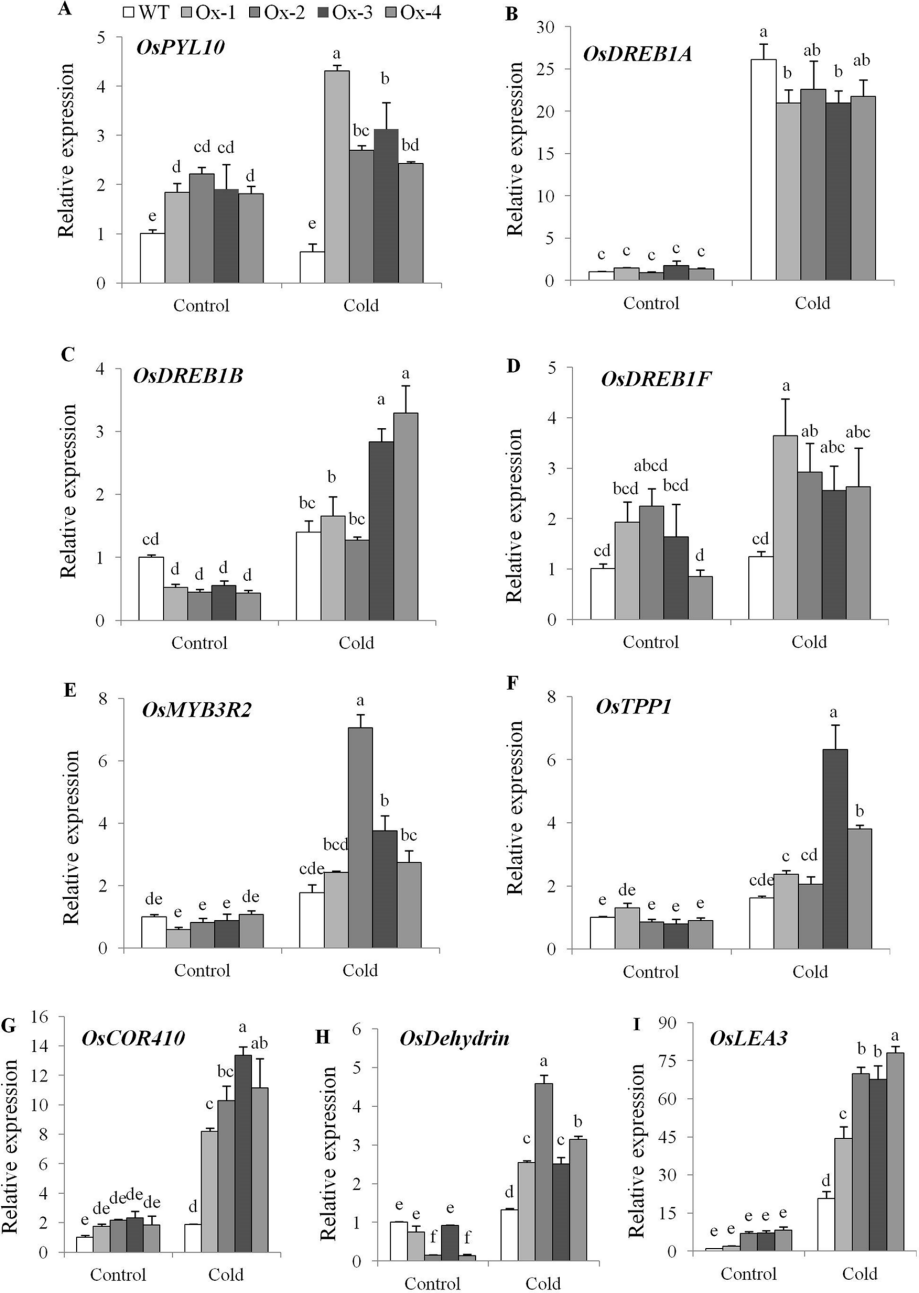
Expression analysis of cold stress responsive genes. **(A)** Expression of *PYL10* under cold stress. **(B**–**I)** qRT-PCR expression analysis of cold stress responsive genes viz., *OsDREB1A*, *OsDREB1B*, *OsDREB1F*, *OsMYB3R2*, *OsTPP1*, *OsCOR410*, *OsDEHYDRIN* and *OsLEA3* under cold stress. Total RNA isolated from leaf tissues of 60 days old plants treated with cold (15°C for 48 h followed by 6°C for 48 h). The experiments were performed with three biological replicates (n = 3). Error bar indicates ± SE. Statistical differences are shown on the bars by labelling significantly different groups with different letters.

Twenty five days old *OsPYL10* overexpressing transgenic (T2 progeny) and WT rice seedlings were cold acclimated at 15°C for 2 day and further exposed to 6°C cold stress for 2 days in a cold room. At the end of cold stress, *OsPYL10* overexpressing lines Ox-2 and Ox-4 showed better membrane stability as compared to WT (**Figures 4A, B**). WT plants did not recover and showed 100% mortality when kept in greenhouse at 30°C for recovery. *OsPYL10* overexpressing transgenic lines showed 16-57% survival (**Figures 4C, D**). PYL10 overexpression lines showed higher expression levels of cold responsive genes viz., *DREB1F*, *MYB3R2*, *TPP1*, *COR410*, *DEHYDRIN*, *and LEA*3 as compared with that of WT plants under cold stress (**Figure 5**).

### *OsPYL10* Confers Cold Stress Tolerance

#### Drought Stress Tolerance at Vegetative Stage

**Figure 6:**
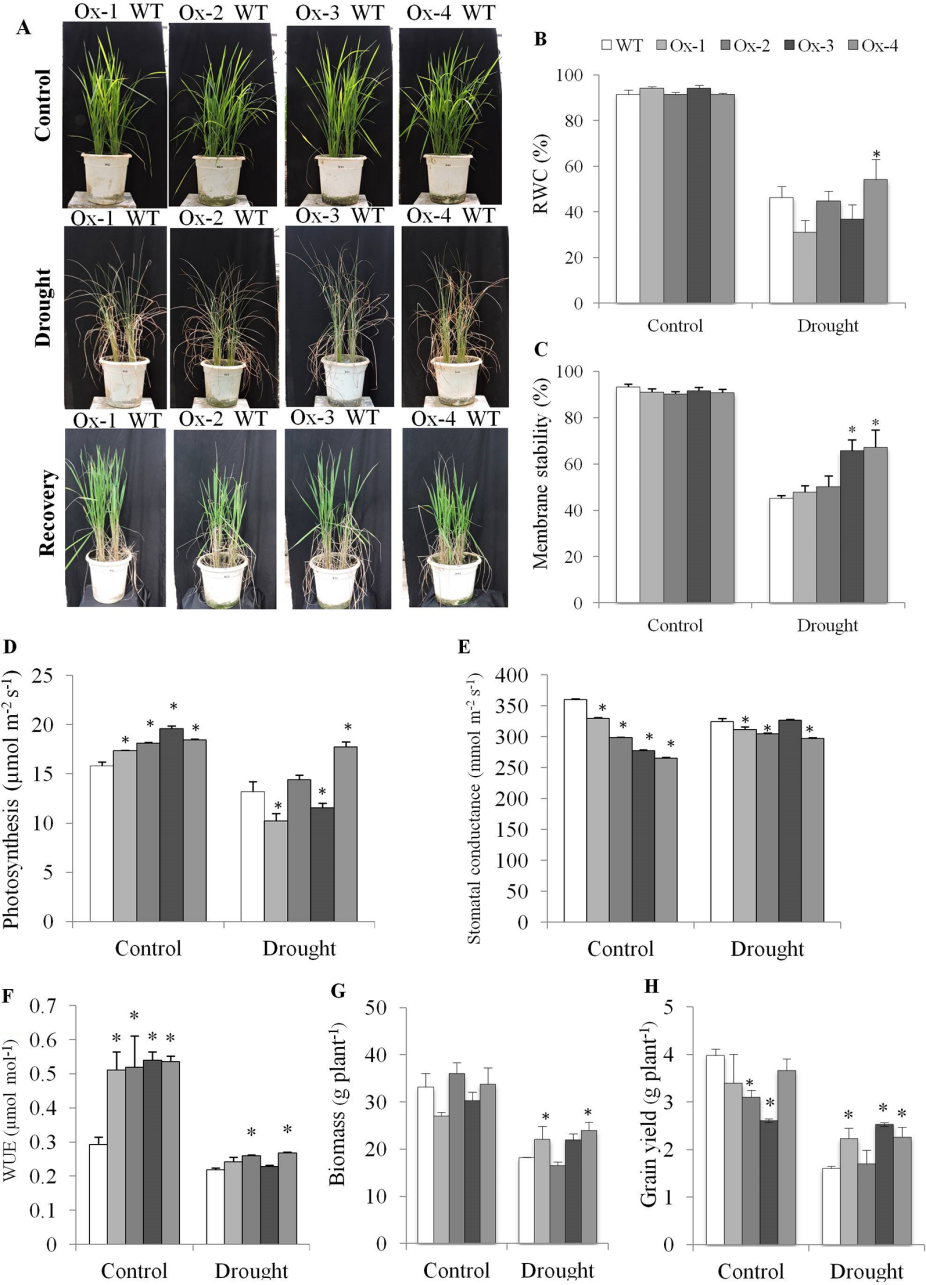
Evaluation of drought tolerance in *OsPYL10* overexpressing transgenic rice at vegetative stage. **(A)** Phenotype of transgenic and WT plants under drought stress and recovery. Drought (∼ -80 kPa) was exposed to transgenic and WT plants by withholding water and recovered by rewatering. **(B**–**E)** Relative water content, membrane stability and photosynthesis of plants under control and drought. **(F**–**H)** WUE, plant biomass and grain yield at maturity of plants control and drought stress. The experiments were performed with three biological replicates (n = 3). Error bar indicates ±SE. *means *P* < 0.05.

WT and transgenic lines were grown in same pot. At vegetative stage, drought stress was imposed by withholding water till the soil matric potential reached up to ∼ -80 kPa (**Figure 6A**). Drought stress reduced the RWC % to about 46% in both WT and overexpression lines except that of Ox-4 which maintained significantly higher RWC (**Figure 6B**). MS (Membrane Stability) decreased to about 45% in WT, while Ox-3 and Ox-4 lines maintained significantly higher membrane stability as compared with WT under drought stress (**Figure 6C**). The rate of photosynthesis was higher in Ox-2 and Ox-3 under control conditions as compared to WT. Drought stress induced reduction in photosynthesis was similar in WT and Ox-1, Ox-2, and Ox-3, while Ox-4 overexpression line showed significantly higher photosynthesis than other genotypes under drought stress (**Figure 6D**). In consistent with high ABA level in transgenics under non-stress conditions, all four transgenic lines showed significantly less stomatal conductance as compared with WT plants (**Figure 6E**). All four transgenic *OsPYL10* overexpressing lines showed significantly higher instantaneous water use efficiency (insWUE) under non-stress conditions, while under drought stress, two overexpression lines (Ox-2 and Ox-4) showed significantly higher insWUE under non-stress conditions as compared with the WT plants (**Figure 6F**).

*PYL10* overexpressing lines produced about the same amount of biomass as WT except Ox-1 which produced slightly lower amount of biomass. Under drought stress, transgenic overexpression lines Ox-1, Ox-2, and Ox-4 produced significantly higher biomass than that of WT plants (**Figure 6G**). Grain yield per plant was similar in WT and Ox-1 and Ox-4 lines, while Ox-2 and Ox-3 lines produced lower grain yield than WT plants under control conditions. However, under drought stress conditions, PYL10 overexpressing transgenics Ox-1, Ox-3 and Ox-4 produced significantly higher grain yield than WT plants (**Figure 6H**). However, the harvest index (ratio of grain yield to total biomass, a measure of photosynthate translocation efficiency and fertility) was similar among the genotypes, and drought stress decreased the harvest index in both WT and transgenic lines.

### Drought Stress Tolerance at Reproductive Stage

**Figure 7:**
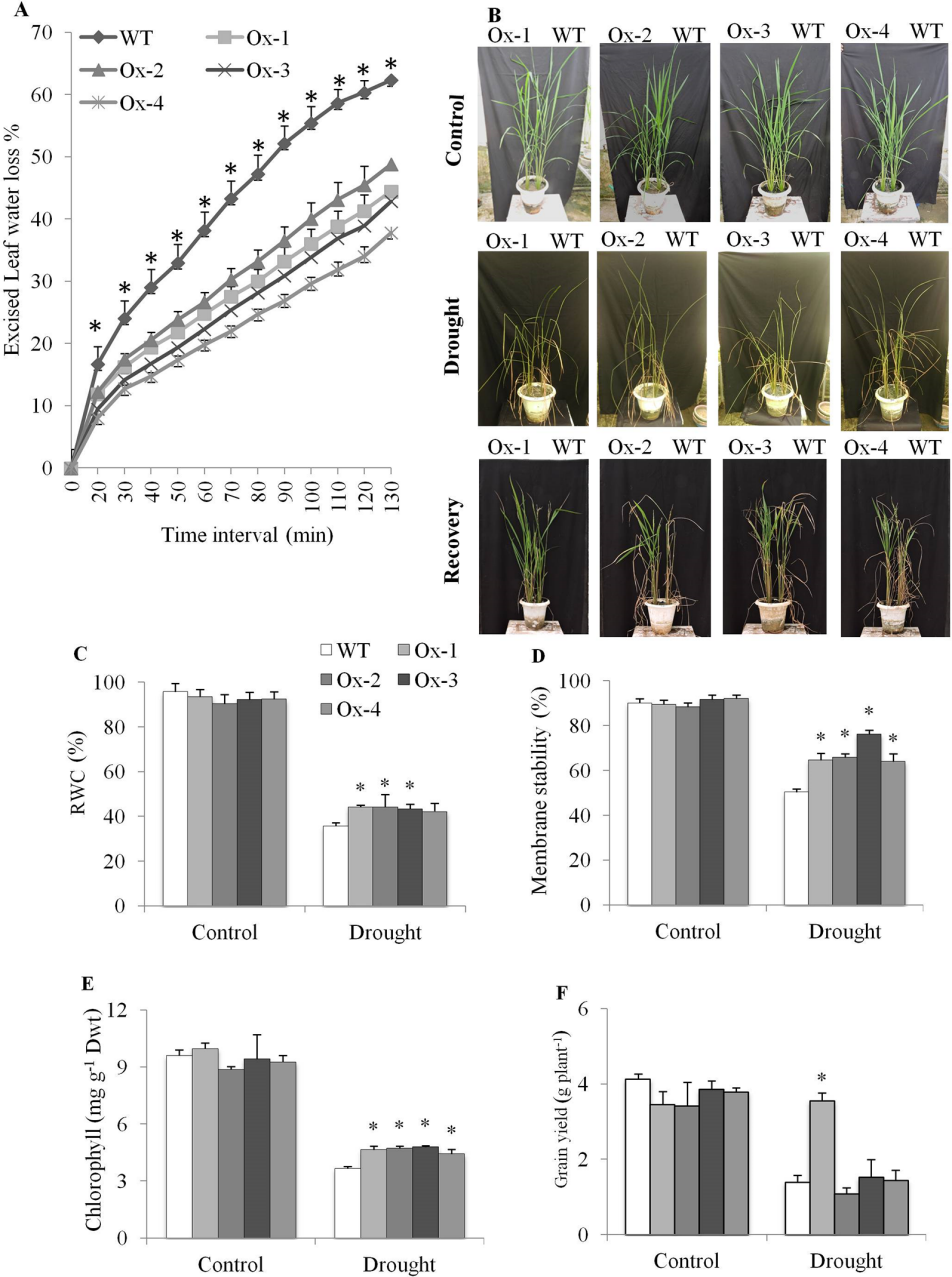
Drought tolerance of *OsPYL10* overexpressing transgenic rice at reproductive stage. **(A)** Excised leaf water loss from excised flag leaves at different time interval of dehydration. The ELWL of transgenic events Ox1, Ox3 and Ox4 were significantly lower than that of WT plants from 20 minutes onwards, while that of Ox2 event was significantly lower than the WT plants from 30 minutes onwards. **(B)** Phenotype of *OsPYL10* transgenic rice and WT plants under drought and recovery. Plants were exposed to drought (∼ -80 kPa) and recovered by irrigation. **(B**–**E)** RWC%, membrane stability, chlorophyll content of transgenic and WT plants under drought stress. Flag leaf was used for all estimation. **(F)** Grain yield under control and drought stress. The experiments were performed with three biological replicates and graph shown the mean value (n = 3). Error bar indicates ± SE. *means *P* < 0.05.

Excised leaf water loss assay was carried out with leaves from well-irrigated plants. WT plants lost about 62% water, while *OsPYL10* overexpressing transgenic lines showed only about 40% water loss at the end 130 min assay period (**Figure 7A**). *OsPYL10* overexpressing transgenic (T_1_ progeny) lines and WT plants were exposed to drought stress at booting stage by withholding water till SMP reached to about ∼ -80 kPa. Soil moisture content was reduced to about 5.8 ± 0.4% in drought pots. PYL10 plants showed better recovery (**Figure 7B**). Drought stress reduced the RWC, membrane stability, and chlorophyll content. However, transgenic lines maintained significantly higher levels of RWC, membrane stability, and chlorophyll content than that of WT plants under drought stress (**Figures 7C–E**). Stress induced H_2_O_2_ accumulation and MDA contents were significantly lower in *PYL10* overexpression lines as compared to that of WT, except for Ox-2 line (**Supplementary Figures S6 A, B**). PYL10 overexpression did not reduce biomass under non-stress and drought stress conditions (**Supplementary Figure S6C**). Overexpression of *PYL10* from *RD29A* promoter did not cause yield penalty. Under drought stress conditions Ox-1 overexpression line produced about 2.5 fold higher grain yield as compared with that of WT (**Figure 7F**).

### Expression of ABA Regulated Genes Under Drought Stress

**Figure 8:**
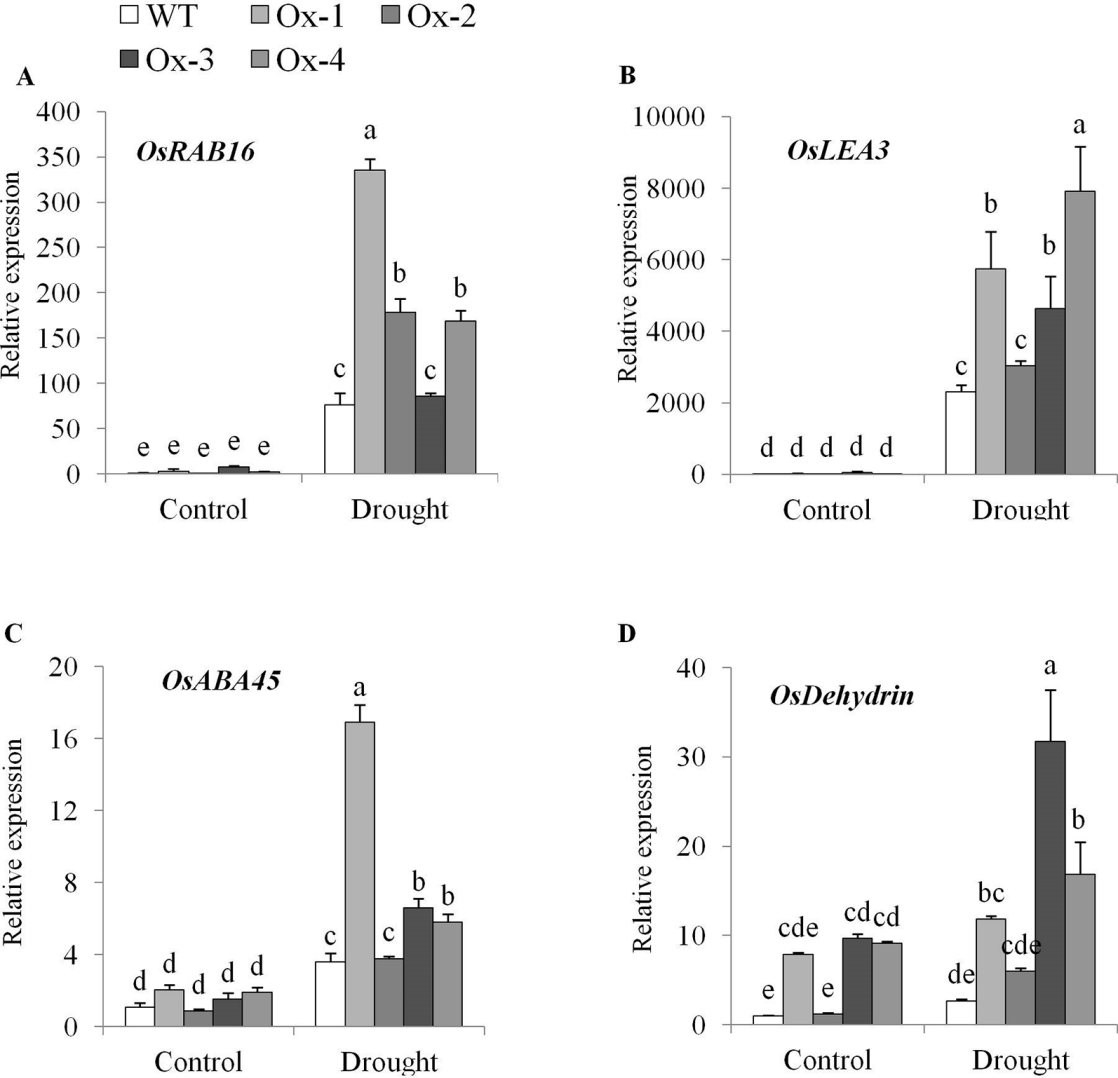
Expression analysis of ABA-dependent pathway genes in OsPYL10 overexpressing transgenic rice and WT plants under control and drought at anthesis. Relative Expression of **(A)** *OsRAB16*, **(B)** *OsLEA3*, **(C)** *OsABA45* and **(D)** *OsDEHYDRIN* genes. Total RNA isolated from leaf tissues of plants under control and drought (∼ -80 kPa) were used for expression analysis by qRT-PCR. Values are mean from three biological replicates (n = 3). Error bar indicates ± SE. Statistical differences are shown on the bars by labelling significantly different groups with different letters.

Gene expression analysis showed significant upregulation of ABA-dependent target genes viz., *OsRAB16*, *OsLEA3A*, *OsABA45*, and *OsDehydrin* in transgenic rice under drought stress as compared with WT plants (**Figures 8A–D**).

## Discussion

In the present study ABA receptor gene *OsPYL10* was functionally validated in transgenic *Indica* rice mega variety MTU1010 by stress inducible overexpression analysis. qRT-PCR expression analysis showed that *OsPYL10* expression was highest in flag leaf of rice (**Figure 1**). Chen et al., 2017 also showed a similar expression pattern for *OsPYL10* in japonica cv. Nipponbare, and thus suggesting similar tissue specific expression of *OsPYL10* across diverse genotypes. Drought stress downregulated the expression of *OsPYL10* in MTU1010 (**Figure 1**). Previous study with rice cv. IR64 (one of the parent of MTU1010) showed that drought stress downregulated the expression of *OsPYL10* in seedlings (Lenka et al., 2018). Overexpression of *bona fide* ABA receptors is expected to confer enhanced sensitivity to ABA. In previous studies, constitutive overexpression (*OsPYL5*, *OsPYL3*, and *OsPYL9*) or endosperm specific overexpression (*OsPYL8* and *OsPYL9*) of rice ABA receptors have been shown to enhance ABA sensitivity during germination and early seedling growth in rice (Kim et al., 2012; He et al., 2014; Tian et al., 2015; Chen et al., 2017). Constitutive overexpression of *OsPYL10* enhanced ABA sensitivity to transgenic Arabidopsis (Lenka et al., 2018). In this study, overexpression of *OsPYL10* from stress inducible *AtRD29A* promoter also moderately enhanced ABA sensitivity during germination (**Supplementary Figure 4**).

Overexpression of *OsPYL10* from stress inducible *AtRD29A* promoter showed only slightly higher level of expression (20 to 80% higher transcript level over WT) in leaves non-stress control conditions (**Figure 2**). Interestingly, leaf ABA content was significantly higher in all four single copy transgenic overexpression lines as compared with WT under control conditions (**Figure 3**). In previous studies, the effect of overexpression of PYLs on ABA content has not been examined. Our study showed that ABA upregulated the expression of *OsPYL10* (**Figure 1**) and overexpression of *PYL10* enhanced ABA accumulation suggesting a potential regulation of leaf ABA content by OsPYL10 ABA receptor in rice. The higher ABA levels in rice *OsPYL10* transgenic lines were associated with constitutively higher expression levels of transgene (*OsPYL10*) and genes for ABA biosynthesis viz., *OsZEP1*, *OsNCED1*, *OsNCED2*, *OsNCED3*, *and OsNCED5* in transgenic lines (**Figure 3**). Previous studies have shown that overexpression of rice *OsNCED3* enhanced ABA content in transgenic Arabidopsis (Hwang et al., 2010) and rice (Huang et al., 2018). It appears that moderately higher levels of *OsPYL10* enhances its ligand ABA production through regulation of genes for ABA synthesis, and may serve as positive feed forward regulation of stress tolerance. The high levels of *OsABA8ox3* (ABA 8’-hydroxylase) in transgenic *PYL10* overexpression lines, but not the *OsBG2* (ABA UDP-glucosyltransferase) (**Figure 3**) suggests that 8’-hydroxylation is a major pathway of ABA catabolism when ABA content is high in rice.

Cold stress affects rice production in temperate regions and in sub-tropical winter season (Sthapit and Witcombe, 1998; Sipaseuth et al., 2007; Fujino and Matsuda, 2010; Ma et al., 2015). Rice has an optimum temperature of about 25–30°C for seedling and vegetative growth. A temperature below 10–13°C cause cold damage during germination and vegetative stage (Yoshida, 1981). In general, *Japonica* accessions are more cold tolerant than *Indica* accessions (Glaszmann et al., 1990; Pan et al., 2015). In an earlier study, constitutive overexpression of *OsPYL3* and *OsPYL9* in *Japonica* cv. Nipponbare conferred tolerance to 10°C for 4 days (Tian et al., 2015). *OsPYL10* when overexpression conferred cold tolerance to Arabidopsis (Lenka et al., 2018). In this study, we showed that stress-inducible overexpression of *OsPYL10* confers seedling stage tolerance to cold stress (6°C for 2 days), at least in part, due to protection of membrane stability under cold stress (**Figure 4**). To further analyze the cold tolerance of *OsPYL10* transgenic lines, previously known ABA-independent and ABA-dependent genes involved in cold tolerance were analyzed. In WT plants *PYL10* expression under 6°C, 48 h cold stress was similar to that of control conditions. However, in transgenic plants *OsPYL10* expression was significantly higher under cold stress as compared to the control suggesting that enhanced induction of *AtRD29A* promoter under cold stress led to the high levels of *OsPYL10* transcripts (**Figure 5A**). Previous study showed *OsDREB1A* is cold induced, but not regulated by ABA (Dubouzet et al., 2003). In our study also cold stress upregulated the expression of *DREB1A* and the induction levels were similar in both WT and *PYL10* overexpression lines (**Figure 5B**). Earlier studies showed that *OsDREB1F* transcription factor is upregulated by ABA (Wang et al., 2008). In our study also, *OsDREB1F* expression was significantly higher in *PYL10* overexpression lines (**Figure 5D**). *OsTPP1* was also shown to be transiently upregulated by ABA and cold stress (Ge et al., 2008). In our study also *OsTPP1* expression was found to be upregulated and the expression levels of *OsTPP1* were higher in *PYL10* overexpression lines as compared with WT plants under cold stress (**Figure 5F**). Expression levels of multiple stress regulated effector genes such as *OsCOR410*, *OsDEHYDRIN*, and *OsLEA3* were upregulated under cold stress, and the expression levels were significantly higher in *PYL10* overexpression lines as compared with WT plants under cold stress (**Figures 5G–I**). These results suggest that expression of *OsDREB1F*, *OsTPP1*, *OsCOR410*, *OsDehydrin*, and *OsLEA3* under cold stress is, at least in part, regulated through PYL ABA receptor signalling pathway. Previous studies have shown that overexpression of *OsDREB1A* (Dubouzet et al., 2003), *OsDREB1B* (Ito et al., 2006), *OsDREB1F* (Wang et al., 2008), *OsMYB3R2* (Ma et al., 2009), and *OsTPP1* (Ge et al., 2008) enhanced tolerance of transgenic plants to cold and other abiotic stresses. In our study also the expression level of *DREB1B*, *DREB1F*, *MYB3R2*, *TPP1*, and *DEHYDRIN* were higher in *OsPYL10* overexpression lines compared to WT under cold stress, and thus might have contributed to the higher cold tolerance of *OsPYL10* transgenic lines. This suggests that ABA signalling pathway plays a key role in cold tolerance of rice, and cold tolerance of indica rice cultivars can be engineered by using *OsPYL10* and other ABA receptors.

Most of the irrigation water used for crop cultivation is used by for paddy rice. Rice cultivation is expected to be unsustainable in future as the per capita water availability is expected to decline by 15 to 54% in most river basins of by the year 2025 in India (Guerra et al., 1998). In India, rainfed lowland and upland rice is cultivated in about 45% of the rice grown area, which are subjected to drought stress. Therefore, genetic improvement in water use efficiency (WUE) and drought tolerance of rice is necessary for food security. Since ABA is the major hormone controlling transpiration and drought tolerance (Finkelstein, 2013), engineering ABA receptors is a potential option to improve WUE and drought tolerance in rice. Previous studies have shown that ABA receptors can be engineered to enhance drought tolerance in different plants including rice (**Table 1**). Kim et al. (2014) showed that constitutive overexpression of *OsPYL5* can increase vegetative drought tolerance by significantly reduced biomass and grain yield. Our study showed that *OsPYL10* overexpression produced similar biomass as compared to that of WT plants under control conditions (**Figure 6** and **Supplementary Figure S6**), and thus suggesting no adverse effect of stress-inducible *AtRD29A* promoter driven overexpression of *OsPYL10*.

As all *PYL10* overexpression lines showed constitutive expression of transgene *PYL10* and produced higher ABA even under non-stress conditions, transgenic lines are expected to have reduced stomatal conductance. In consistent with this, all transgenic lines showed significantly less stomatal conductance under non-stress conditions, but photosynthesis was significantly higher (**Figures 6D, E**), suggesting that WUE under control conditions can be enhanced by overexpression of *OsPYL10*. Previous study with overexpression of Arabidopsis ABA receptors *RCAR6*/*PYL12* and *RCAR10*/*PYL4* showed that these receptors confer enhanced WUE to transgenic Arabidopsis plants (Yang et al., 2019). In consistent with this, the insWUE of *OsPYL10* overexpressing transgenic rice lines were significantly higher under non-stress conditions (**Figure 6F**).

Of the two experiments conducted, grain yield of *OsPYL10* transgenic under control conditions was similar to WT except for overexpression lines Ox-2 and Ox-3 in one of the experiment (**Figure 6** and **Supplementary Figure S6**). In vegetative stress experiments, *OsPYL10* overexpression lines Ox-1, Ox-3, and Ox-4 produced 40–58% higher grain yield as compared with WT (**Figure 6**) while under reproductive stage drought stress only one overexpression line Ox-1 produced > two-fold higher grain yield (**Supplementary Figure S6**). These results suggests that stress regulated overexpression of ABA receptors is useful strategy to enhance grain yield under drought stress while maintaining productivity under non-stress conditions.

On the component mechanisms of drought tolerance, overexpression of ABA receptors viz., *OsPYL3*, *OsPYL5*, and *OsPYL9* has been shown to reduce excise leaf water loss in transgenic rice (Kim et al., 2014; Tian et al., 2015). In this study also, stress-inducible overexpression of *OsPYL10* minimised excised leaf water loss significantly as compared with WT plant leaves (**Figure 7**). In Arabidopsis, overexpression of one of the ABA receptor *AtPYL9* has been shown to enhance drought tolerant by reducing transpiration and promoting leaf senescence under drought stress (Zhao et al., 2016). However, in our study, *OsPYL10* overexpression did not induce leaf senescence, but protected chlorophyll content under drought stress (**Figure 7**). This may be due to the difference in the mechanism of action of different receptors in conferring drought tolerance.

In this study, stress inducible overexpression of *OsPYL10* resulted in significantly higher expression levels of ABA-dependent pathway genes viz., *OsRAB16*, *OsABA45*, *OsLEA3*, and *OsDEHYDRIN* in transgenic plants as compared to WT plants under drought stress (**Figure 8**). Previously, overexpression of ABA receptor *OsPYL3*, *OsPYL5*, and *OsPYL9* has been shown to enhance the expression of these genes in rice (Kim et al., 2014; Tian et al., 2015).

## Conclusion

Overexpression of *OsPYL10* enhanced ABA accumulation through upregulation of gene for ABA synthesis, and thus enhanced instantaneous WUE and minimized excised leaf water loss in rice. Since both WT and transgenic plants grown in same pot were subjected to drought stress, the soil moisture stress was same for both the transgenics and WT plants. Therefore, ABA mediated cellular stress tolerance appears to be the major contributing mechanism of stress tolerance of transgenic plants. This evident from the significant upregulation of *OsRAB16*, *OsABA45*, *OsLEA3*, and *OsDEHYDRIN* genes in transgenic plants under drought stress. In addition, it is also possible that PYL10 mediated efficient soil moisture extraction through regulation of aquaporin and osmotic adjustment might have also contributed for tolerance to *PYL10* transgenic plants. Overexpression of *PYL10* also upregulated the expression of cold responsive genes viz., *OsDREB1B*, *OsDREB1F*, *OsMYB3R2*, *OsTPP1*, *OsDEHYDRIN*, and *OsLEA*3 and thus, enhanced the cold tolerance of *indica* rice. Since stress-inducible overexpression of *OsPYL10* gene is not detrimental to plant growth and yield under non-stress conditions, but protects yield under drought stress conditions, PYL10 can be useful to enhance both cold tolerance and drought tolerance in rice plants. Functional analysis of all the ABA receptor family members will help design climate resilient rice crop with “*more crop per drop*” and multiple abiotic stress tolerance.

## Data Availability Statement

The datasets generated for this study can be found in the GenBank: KF925265.1.

## Author Contributions

SY cloned the gene and did rice transformation. RV managed the plant material and molecular analyses. RV and VS did physiological analysis for drought and cold stress experiment and expression analysis. SP did the ABA estimation. MR helped in project preparation and data analysis. VC designed the project and supervised the project. VC and RV designed the experiment, analyzed the data, and wrote the manuscript. All authors read and approved the manuscript.

## Funding

This work was funded by NASF (ICAR) grant no. NFBSFARA/Phen-2015 and IARI inhouse project grant no. CRSCIARISIL20144047279.

## Conflict of Interest

The authors declare that the research was conducted in the absence of any commercial or financial relationships that could be construed as a potential conflict of interest.
