# Supplementary material for: Overexpression of ABA Receptor PYL10 Gene Confers Drought and Cold Tolerance to Indica Rice
Source: Front Plant Sci. 2019 Nov 28;10:1488. doi: 10.3389/fpls.2019.01488 (PMC6892954; doi:10.3389/fpls.2019.01488)
Supplement: Supplementary file 1 [file DataSheet_1.docx]

**Supplementary Table 1.** Nomenclature of rice ABA receptors followed in different study

| **RGAP Locus ID** | **Miao et al. 2018** | **Tian et al. 2015** | **He et al. 2014** | **Kim *et al.* 2012** | **No. of Amino acids** | **Introns in gene** | **Apo-receptor form (He et al. 2014)** | **Sub family** |
| --- | --- | --- | --- | --- | --- | --- | --- | --- |
| Os10g42280 | *PYL1* | *PYL10* | *PYL1* | *RCAR10* | 212 | 0 | **Dimer** | **III** |
| Os06g36670 | *PYL2* | *PYL9* | *PYL2* | *RCAR9* | 207 | 0 | **Dimer** | **III** |
| Os02g13330 | *PYL3* | *PYL2* | *PYL3* | *RCAR2* | 207/211 | 0 | **Dimer** | **III** |
| Os01g61210 | *PYL4* | *PYL1* | *PYL4* | *RCAR1* | 208/257 | 0 | Monomer | **II** |
| Os05g39580 | *PYL5* | *PYL6* | *PYL5* | *RCAR6* | 217/216 | 0 | Monomer | **II** |
| Os03g18600 | *PYL6* | *PYL4* | *PYL6* | *RCAR4* | 229 | 0 | **Mono-Dimer** | **II** |
| Os06g33480 | *PYL7* |  | *PYL7* |  | 146/197 | 2 | Monomer | **I** |
| Os02g15640 | *PYL10* | *PYL3* | *PYL10* | *RCAR3* | 204/206 | 2 | Monomer | **I** |
| Os06g33640 | *PYL8* | *PYL7* | *PYL8* | *RCAR7* | 206 | 2 | Monomer | **I** |
| Os06g33690 | *PYL9* | *PYL8* | *PYL9* | *RCAR8* | 206/204 | 2 | Monomer | **I** |
| Os05g12260 | *PYL11* | *PYL5* | *PYL11* | *RCAR5* | 209 | 2 | Monomer | **I** |
| Os02g15620 | *PYL12* |  | *PYL12* |  | 150/181 | 1 | Monomer | **I** |
| Os06g33490 | *PYL13* |  |  |  | 158 | 2 |  |  |

**Supplementary Table 2.** Primers used for TA and binary cloning of *OsPYL10* gene

| **S. No.** | **Name** | **Sequence (5’-3’)** | **Length (bp)** |
| --- | --- | --- | --- |
| 1 | OsPYL10-F | GTTCTTGAGGATGGTGGAGGTG | 22 |
| 2 | OsPYL10-R | TCTTGGAATCTTGGCAACAATCAC | 24 |
| 3 | OsPYL10-F | CGGGATCCGTTCTTGAGGATGGTGGAGGTG | 30 |
| 4 | OsPYL10-R | CCGAGCTCTTGGAATCTTGGCAACAATCAC | 30 |

**Supplementary Table 3.** Primers used for qRT-PCR

| **S. No.** | **Name** | **Sequence (5’-3’)** | **Length (bp)** |
| --- | --- | --- | --- |
| 1 | OsPYL10q-F | CAAGGATGAGACATGCTACTTCGT | 20 |
| 2 | OsPYL10q-R | AGGCAGGCCAGACTTAACAT | 20 |
| 3 | OsRAB16A-F | CAACGCTCCGGCAGCTCCAG | 20 |
| 4 | OsRAB16A-R | ATGCTGCTGCTCGCCCTTGT | 20 |
| 5 | OsLEA3-F | AGACCTCCAGCACGTCGCAG | 20 |
| 6 | OsLEA3-R | GCCTGTTGGAGGACGCTGCC | 20 |
| 7 | OsABA45-F | AGAGAGGGGACAGCCCGATG | 20 |
| 8 | OsABA45-R | AGGCTCAGCTTCCCCATCGC | 20 |
| 9 | OsDehyd-F | GGAGGAGTTCGTAGCAGGAT | 20 |
| 10 | OsDehyd-R | CGTGCAGCCATTATTATCAG | 20 |
| 11 | OsNCED1-F | CTCACCATGAAGTCCATGAGGCTT | 24 |
| 12 | OsNCED1-R | GTTCTCGTAGTCTTGGTCTTGGCT | 23 |
| 13 | OsNCED2-F | GGTATGGAAACGAGGATAGTGGTT | 25 |
| 14 | OsNCED2-R | TGCTTATTGTTGTGCGAGAAGTTC | 24 |
| 15 | OsNCED3-F | CCCTCCCAAACCATCCAAACCGA | 23 |
| 16 | OsNCED3-R | GTGAGCATATCCTGGCGTCGTGA | 23 |
| 17 | OsNCED4-F | TCCATCTCCTTCTCCCTCCTCCCA | 23 |
| 18 | OsNCED4-R | CCTCGCACCCTGCTTGATCTTGCC | 24 |
| 19 | OsNCED5-F | ACATCCGAGCTCCTCGTCGTGAA | 24 |
| 20 | OsNCED5-R | TTGGAAGGTGTTTTGGAATGAACCA | 25 |
| 21 | OsABA8ox3-F | AGTACAGCCCATTCCCTGTG | 20 |
| 22 | OsABA8ox3-R | ACGCCTAATCAAACCATTGC | 20 |
| 23 | OsZEP-F | GATGATGGCAACAAGGTAACTGCA | 24 |
| 24 | OsZEP-F | CTGTGTCAATGTCAGGAGGCAC | 22 |
| 25 | OsBG2-F | GTCATGGAATGTCAGGCAAGGAG | 23 |
| 26 | OsBG2-F | CTCTTCAGTCCATCCTTGTAGTC | 23 |
| 27 | OsDREB1B-F | TGCCTCAACTTCGCCGACTTC | 21 |
| 28 | OsDREB1B-R | TGGCTTCTTCTTCGTCGCCAT | 21 |
| 29 | OsDREB1F-F | ATCCGCAGGAAGGCACGAGC | 21 |
| 30 | OsDREB1F-R | TCATCGTCGTCGTCGATGGC | 20 |
| 31 | OsDREB1A-F | TGAGTGACATGGGCTGGGACC | 21 |
| 32 | OsDREB1A-R | TGGCATCGGAAGCCAGAAAAG | 21 |
| 33 | OsCOR410-F | GCCTCTTCGACAACCTCCTT | 20 |
| 34 | OsCOR410-R | CCTCCTTCTTTGGCTCTTCC | 20 |
| 35 | OsTPP1-F | TGTCGCCTATTGTGGATGAT | 21 |
| 36 | OsTPP1-R | ACACCTTATTGCGGGACCTT | 21 |
| 37 | OsMYB3R2-F | CAGGGTTTCTATCTCGTTCC | 21 |
| 38 | OsMYB3R2-R | ATTTCCAAGCCCTTACCAC | 19 |
| 39 | Nos-R | TACATGCTTAACGTAATTCAACAG | 24 |
| 40 | OsUBQ5-F | CCAGGACAAGATGATCTGCC | 20 |
| 41 | OsUBQ5-R | AAGAAGCTGAAGCATCCAGC | 20 |
