# Supplementary material for: Overexpression of ABA Receptor PYL10 Gene Confers Drought and Cold Tolerance to Indica Rice
Source: Front Plant Sci. 2019 Nov 28;10:1488. doi: 10.3389/fpls.2019.01488 (PMC6892954; doi:10.3389/fpls.2019.01488)
Supplement: Supplementary file 2 [file Presentation_1.pptx]

## Slide 1
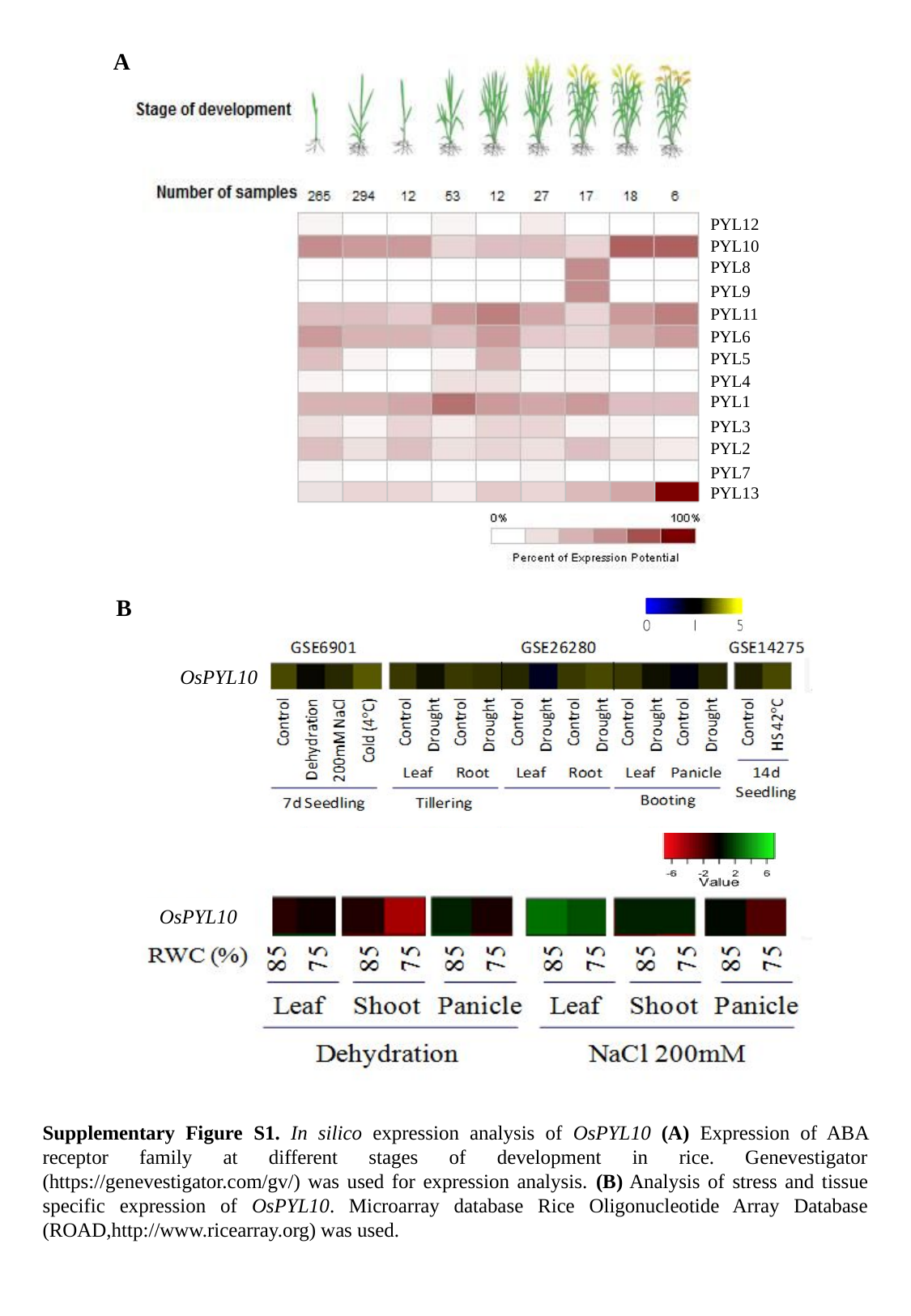

A
PYL12
PYL10
PYL8
PYL9
PYL11
PYL6
PYL5
PYL4
PYL1
PYL3
PYL2
PYL7
PYL13
B
OsPYL10
OsPYL10
Supplementary Figure S1. In silico expression analysis of OsPYL10 (A) Expression of ABA receptor family at different stages of development in rice. Genevestigator (https://genevestigator.com/gv/) was used for expression analysis. (B) Analysis of stress and tissue specific expression of OsPYL10. Microarray database Rice Oligonucleotide Array Database (ROAD,http://www.ricearray.org) was used.

## Slide 2
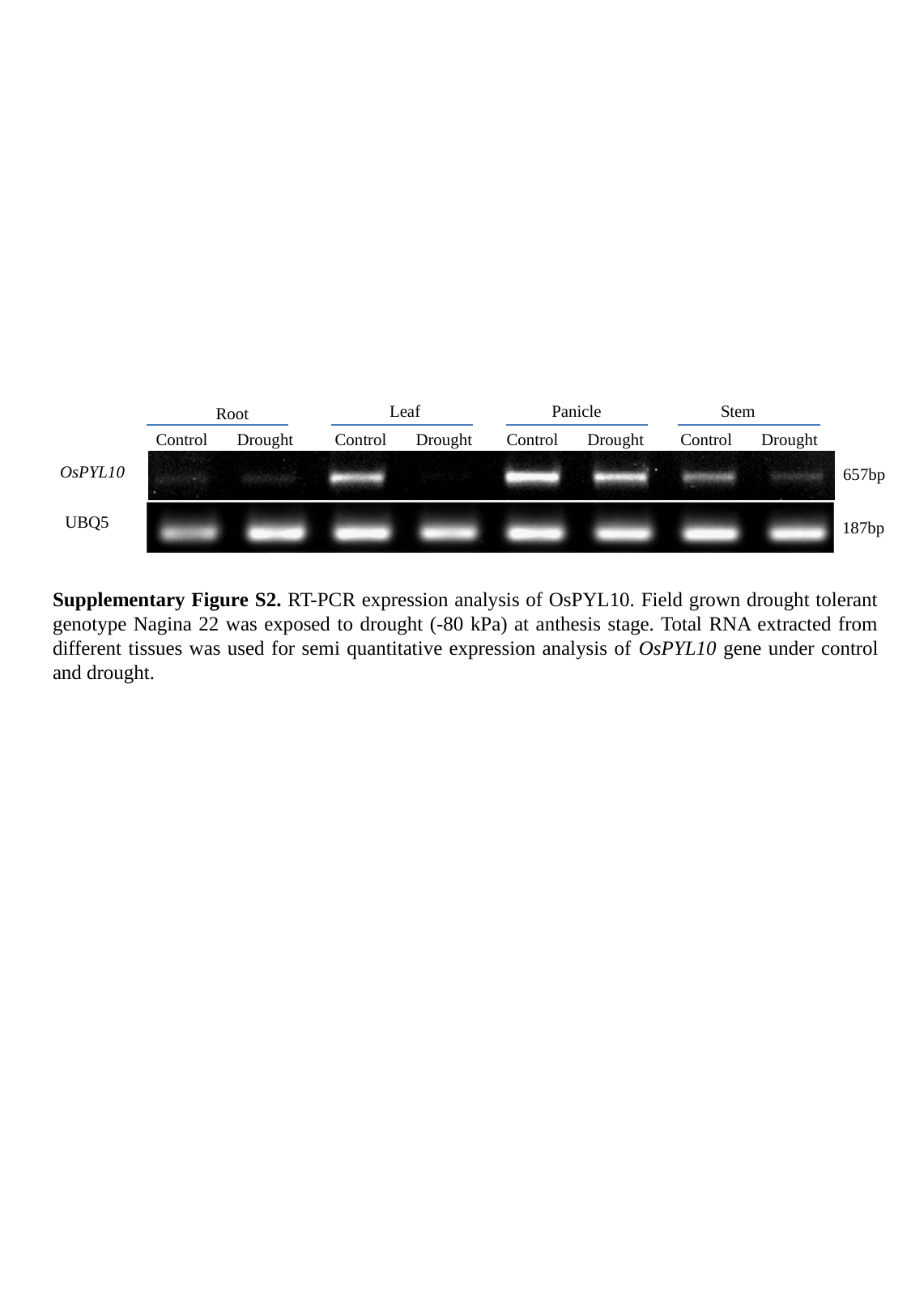

Leaf
Panicle
Stem
Root
Control
Control
Control
Control
Drought
Drought
Drought
Drought
OsPYL10
UBQ5
657bp
187bp
Supplementary Figure S2. RT-PCR expression analysis of OsPYL10. Field grown drought tolerant genotype Nagina 22 was exposed to drought (-80 kPa) at anthesis stage. Total RNA extracted from different tissues was used for semi quantitative expression analysis of OsPYL10 gene under control and drought.

## Slide 3
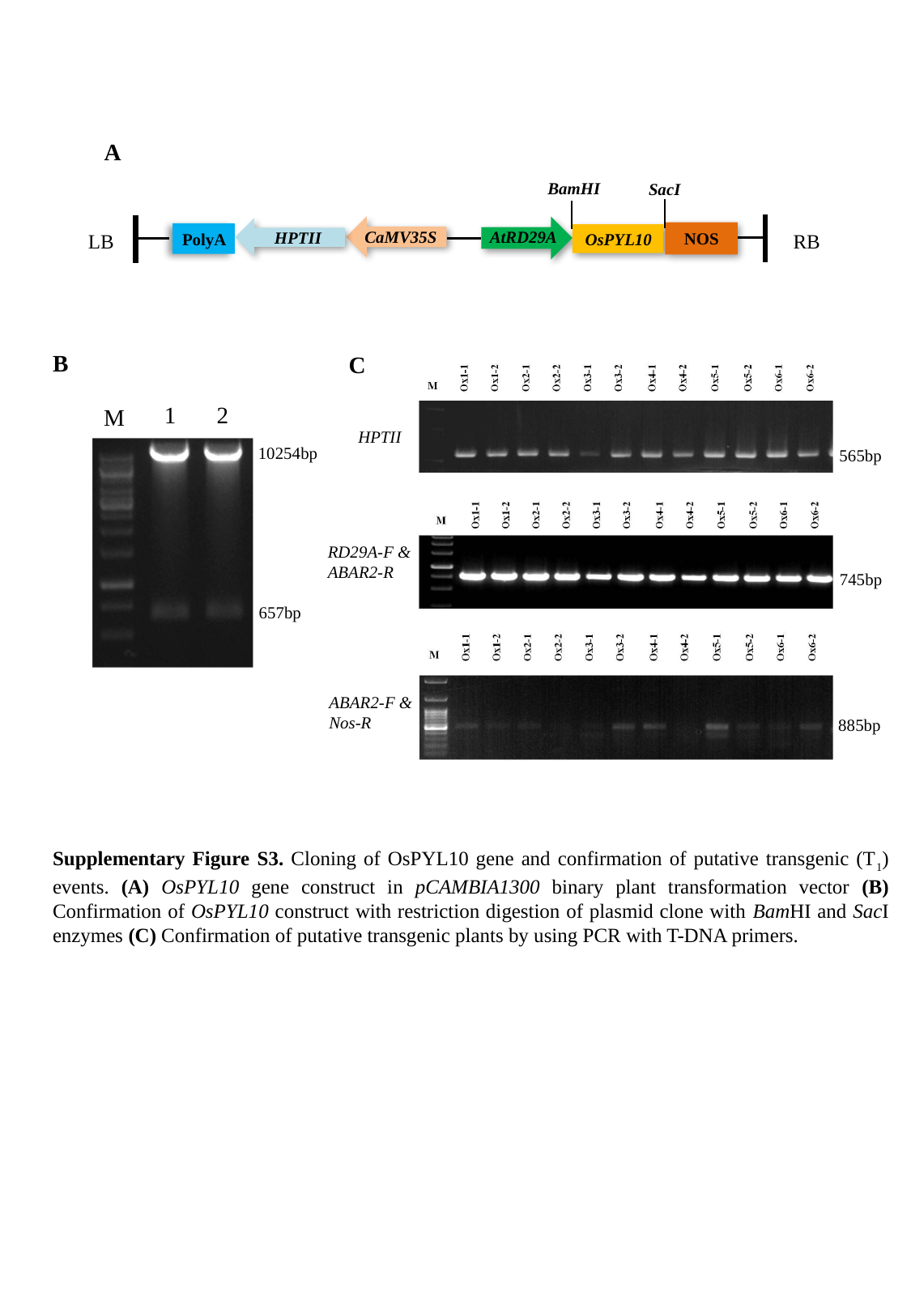

A
BamHI
SacI
AtRD29A
CaMV35S
HPTII
NOS
PolyA
LB
RB
OsPYL10
B
1
2
M
10254bp
657bp
C
HPTII
RD29A-F & ABAR2-R
ABAR2-F & Nos-R
565bp
745bp
885bp
Supplementary Figure S3. Cloning of OsPYL10 gene and confirmation of putative transgenic (T1) events. (A) OsPYL10 gene construct in pCAMBIA1300 binary plant transformation vector (B) Confirmation of OsPYL10 construct with restriction digestion of plasmid clone with BamHI and SacI enzymes (C) Confirmation of putative transgenic plants by using PCR with T-DNA primers.

## Slide 4
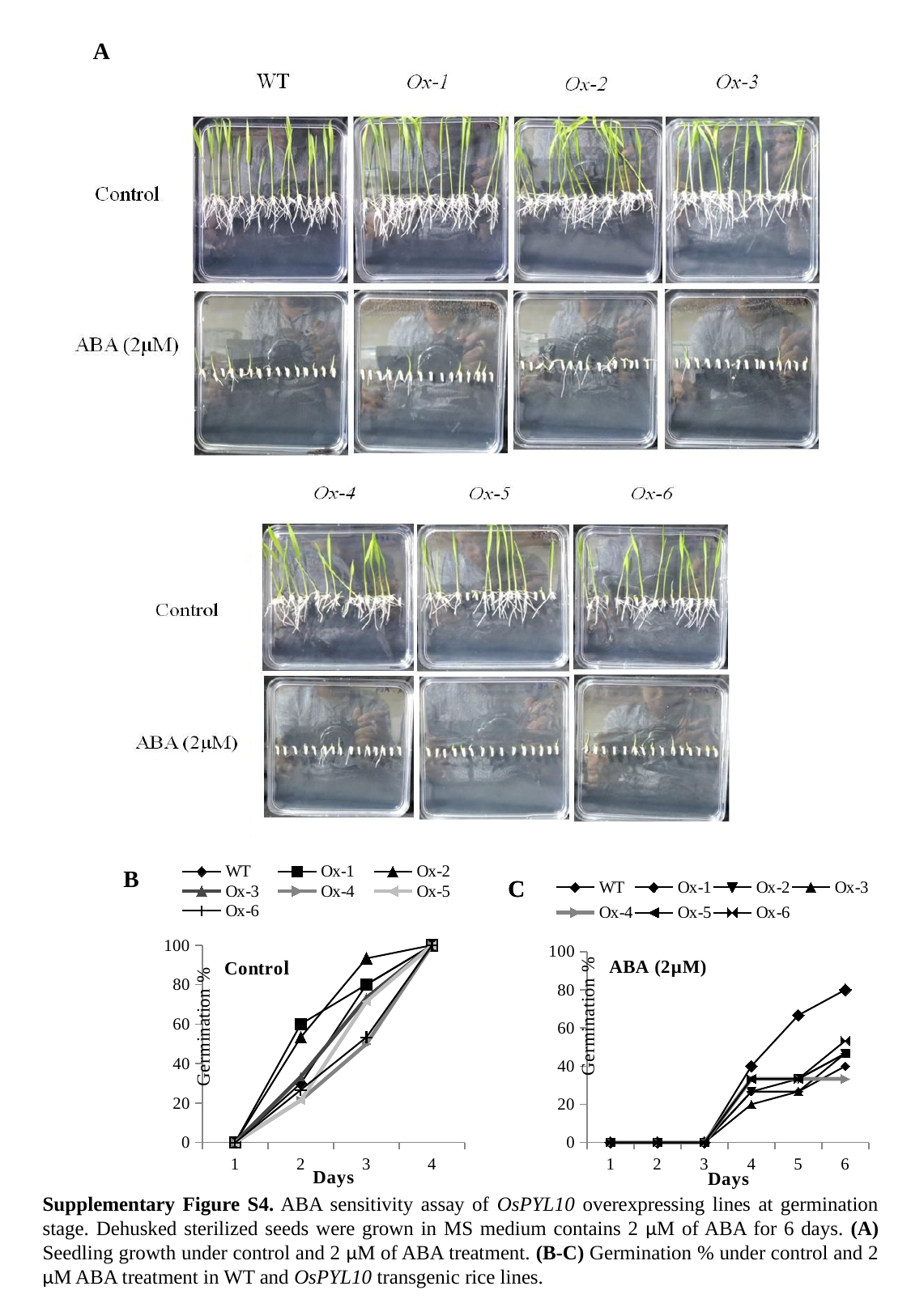

A
### Chart
| Category | WT | Ox-1 | Ox-2 | Ox-3 | Ox-4 | Ox-5 | Ox-6 |
|---|---|---|---|---|---|---|---|
### Chart
| Category | WT | Ox-1 | Ox-2 | Ox-3 | Ox-4 | Ox-5 | Ox-6 |
|---|---|---|---|---|---|---|---|Supplementary Figure S4. ABA sensitivity assay of OsPYL10 overexpressing lines at germination stage. Dehusked sterilized seeds were grown in MS medium contains 2 μM of ABA for 6 days. (A) Seedling growth under control and 2 μM of ABA treatment. (B-C) Germination % under control and 2 μM ABA treatment in WT and OsPYL10 transgenic rice lines.

## Slide 5
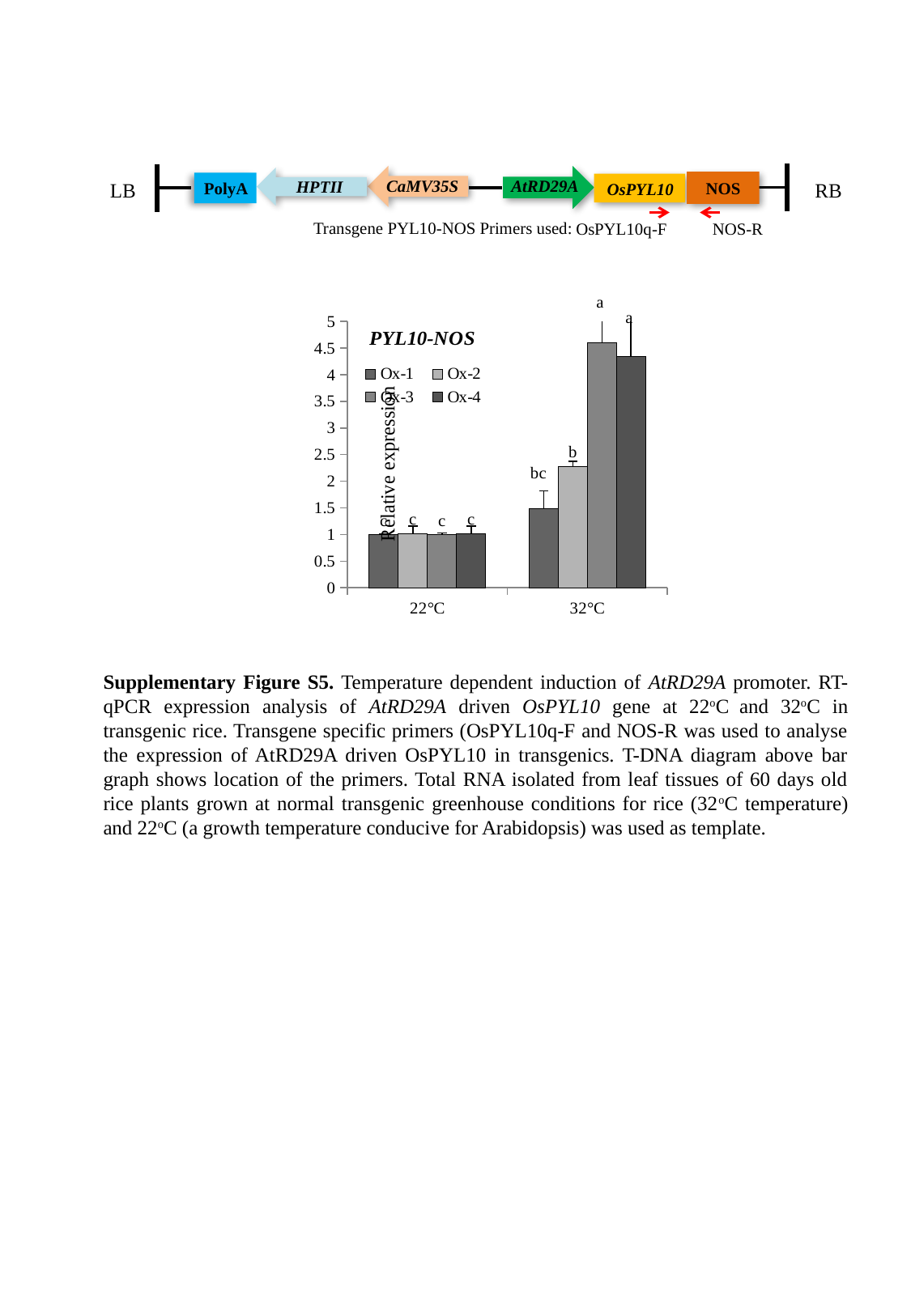

AtRD29A
CaMV35S
HPTII
NOS
PolyA
LB
RB
OsPYL10
Transgene PYL10-NOS Primers used:
OsPYL10q-F
NOS-R
### Chart
| Category | Ox-1 | Ox-2 | Ox-3 | Ox-4 |
|---|---|---|---|---|
| 22°C | 1.0 | 1.02 | 1.0 | 1.02 |
| 32°C | 1.49 | 2.28 | 4.6 | 4.34 |Supplementary Figure S5. Temperature dependent induction of AtRD29A promoter. RT-qPCR expression analysis of AtRD29A driven OsPYL10 gene at 22oC and 32oC in transgenic rice. Transgene specific primers (OsPYL10q-F and NOS-R was used to analyse the expression of AtRD29A driven OsPYL10 in transgenics. T-DNA diagram above bar graph shows location of the primers. Total RNA isolated from leaf tissues of 60 days old rice plants grown at normal transgenic greenhouse conditions for rice (32oC temperature) and 22oC (a growth temperature conducive for Arabidopsis) was used as template.

## Slide 6
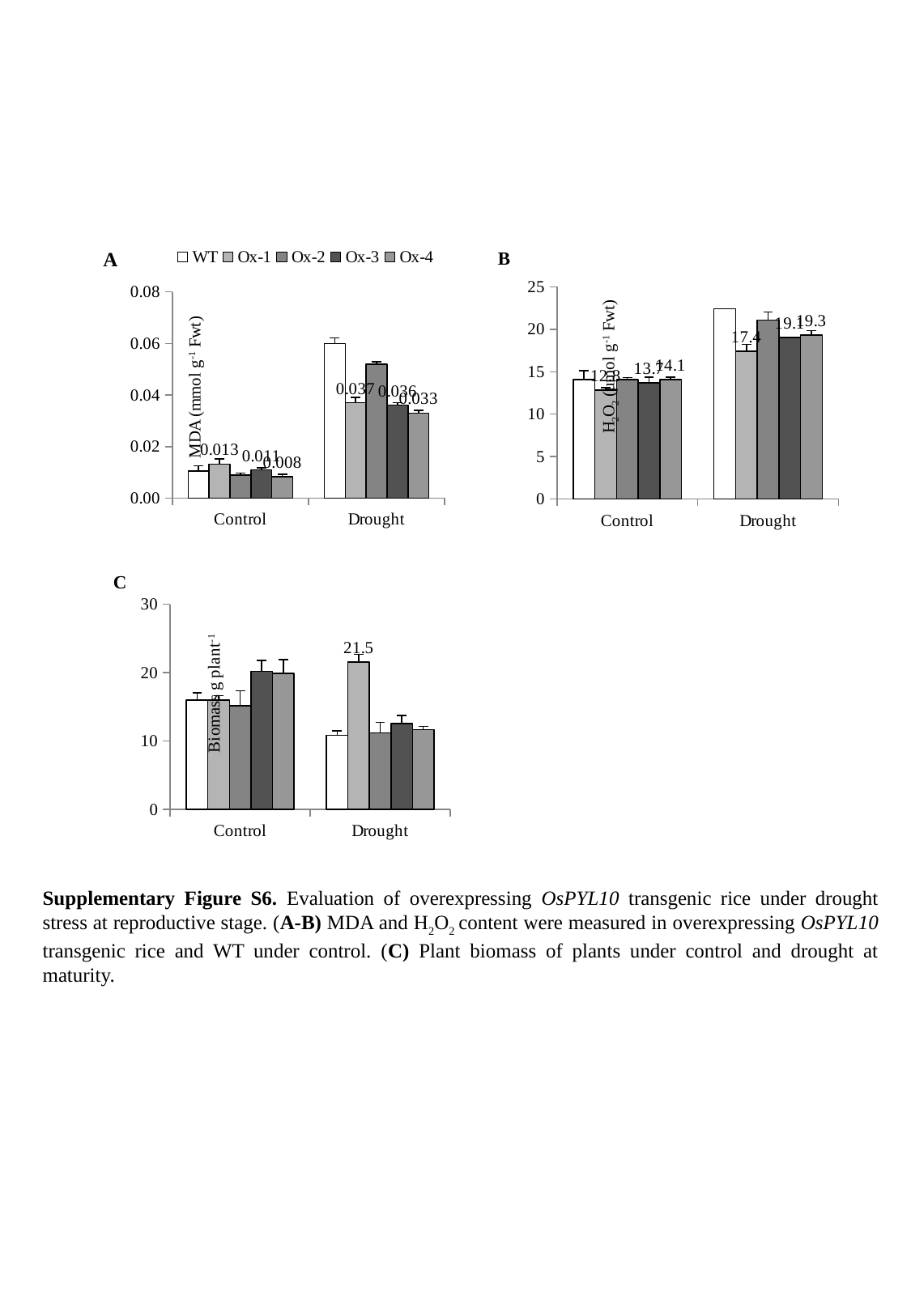

### Chart
| Category | WT | Ox-1 | Ox-2 | Ox-3 | Ox-4 |
|---|---|---|---|---|---|
| Control | 0.010494623655913981 | 0.013161290322580645 | 0.00886021505376352 | 0.0109247311827957 | 0.008344086021505425 |
| Drought | 0.06000000000000003 | 0.037 | 0.052 | 0.036 | 0.033 |
### Chart
| Category | WT | Ox-1 | Ox-2 | Ox-3 | Ox-4 |
|---|---|---|---|---|---|
| Control | 14.090909090909102 | 12.803030303030306 | 14.090909090909102 | 13.712121212121216 | 14.090909090909102 |
| Drought | 22.392676767676775 | 17.424242424242433 | 21.060606060606062 | 19.053030303030287 | 19.31818181818182 |
### Chart
| Category | WT | Ox-1 | Ox-2 | Ox-3 | Ox-4 |
|---|---|---|---|---|---|
| Control | 15.975555555556102 | 15.993333333333332 | 15.176666666666724 | 20.186666666666667 | 19.89333333333249 |
| Drought | 10.817777777777778 | 21.54333333333209 | 11.163333333333332 | 12.520000000000001 | 11.643333333333333 |Supplementary Figure S6. Evaluation of overexpressing OsPYL10 transgenic rice under drought stress at reproductive stage. (A-B) MDA and H2O2 content were measured in overexpressing OsPYL10 transgenic rice and WT under control. (C) Plant biomass of plants under control and drought at maturity.
